# Efficient X-ray luminescence imaging with ultrastable and eco-friendly copper(I)-iodide cluster microcubes

**DOI:** 10.1038/s41377-023-01208-0

**Published:** 2023-06-25

**Authors:** Yanze Wang, Wenjing Zhao, Yuanyuan Guo, Wenbo Hu, Chenxi Peng, Lei Li, Yuan Wei, Zhongbin Wu, Weidong Xu, Xiyan Li, Yung Doug Suh, Xiaowang Liu, Wei Huang

**Affiliations:** 1 Frontiers Science Centre for Flexible Electronics (FSCFE), MIIT Key Laboratory of Flexible Electronics (KLoFE), Shaanxi Key Laboratory of Flexible Electronics, Xi’an Key Laboratory of Flexible Electronics, Xi’an Key Laboratory of Biomedical Materials & Engineering, Xi’an Institute of Flexible Electronics, Institute of Flexible Electronics (IFE), Northwestern Polytechnical University, Xi’an, 710072 Shaanxi China; 2 Key Laboratory of Magnetic Materials Devices, Ningbo Institute of Materials Technology and Engineering, Chinese Academy of Sciences, Ningbo, 315201 China; 3 Institute of Photoelectronic Thin Film Devices and Technology, Solar Energy Conversion Center, Nankai University, Tianjin, 300350 China; 4 Department of Chemistry and School of Energy and Chemical Engineering, UNIST, Ulsan, 44919 Korea; 5 State Key Laboratory of Organic Electronics and Information Displays & Institute of Advanced Materials(IAM), Nanjing University of Posts & Telecommunications, 9 Wenyuan Road, Nanjing, 210023 China; 6 Key Laboratory of Flexible Electronics (KLOFE), Institute of Advanced Materials (IAM), Nanjing Tech University (Nanjing Tech), 30 South Puzhu Road, Nanjing, 211816 China

**Keywords:** X-rays, Optical materials and structures

## Abstract

The advancement of contemporary X-ray imaging heavily depends on discovering scintillators that possess high sensitivity, robust stability, low toxicity, and a uniform size distribution. Despite significant progress in this field, the discovery of a material that satisfies all of these criteria remains a challenge. In this study, we report the synthesis of monodisperse copper(I)-iodide cluster microcubes as a new class of X-ray scintillators. The as-prepared microcubes exhibit remarkable sensitivity to X-rays and exceptional stability under moisture and X-ray exposure. The uniform size distribution and high scintillation performance of the copper(I)-iodide cluster microcubes make them suitable for the fabrication of large-area, flexible scintillating films for X-ray imaging applications in both static and dynamic settings.

## Abstract (web summary)

Monodisperse high-performance scintillating microcubes consisting of copper-iodide clusters were developed for creating a flexible X-ray imaging screen suitable for static and dynamic settings when doped into a polymer matrix.

## Graphical abstract

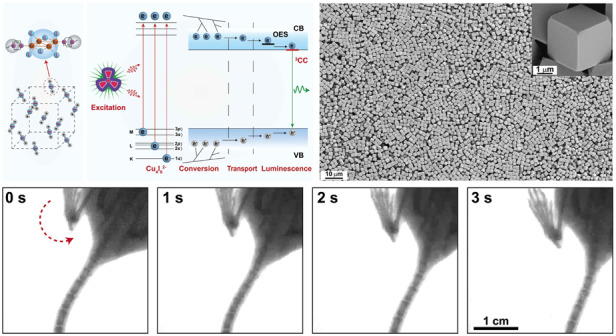

## Introduction

Scintillators are optical materials that emit low-energy ultraviolet and visible photons in response to ionizing radiation such as X-rays and gamma rays^1–7^. This property makes scintillating materials useful for applications like nondestructive testing, X-ray astronomy, security inspection, and medical imaging^8–11^. Traditional inorganic scintillators that contain heavy metals usually have excellent performance, but their high-temperature requirement for bulk crystal growth hampers their use in the development of large-area and flexible X-ray detectors^12–15^. Additionally, many commercially available scintillators like CsI:Tl and LaBr_3_:Ce are hygroscopic, adding difficulty to device fabrication. Recent developments in metal halide nanocrystals, such as CsPbBr_3_ and Cs_4_PbBr_6_, show promise as a new class of scintillators with improved performance that can be processed in solution^16–20^. These features enable the fabrication of high-efficiency flexible X-ray imaging devices based on nanoscintillator-doped plastic substrates^21–23^. However, the challenge remains to develop efficient nano- and micro-scintillators with uniform morphology, environmentally-friendly composition, robust chemical stability, and integration into a stretchable substrate for flexible X-ray detectors^24–26^.

To tackle the challenges outlined above, two key factors must be addressed. The first is to increase both the X-ray absorption ability and conversion efficiency of high-energy X-rays into low-energy photons in a scintillating material^27–30^. The second is to control the crystal growth process to produce homogeneous, small scintillators that are easy to use in flexible scintillation films^31^. Inspiration comes from the structure of a Cu(I)-I cluster, which is made up of a heavy inorganic core and organic ligands^32^. The idea is to create high-performing, eco-friendly scintillators by assembling these building blocks into crystalline nanoscale and microscale crystals^33,34^. This is because the large effective atomic number of Cu(I)-I cluster compounds provides strong X-ray stopping power (Supplementary Eqs. (S1) and (S2)), and the exceptional X-ray conversion efficiency stems from their photoluminescence and semiconducting properties (Supplementary Eq. (S3))^35,36^. Additionally, structure engineering improves the lattice stability of Cu(I)-I cluster scintillators and makes them more resistant to moisture, making it possible to rationally grow the crystals through a wet chemical process.

In this report, we present the development of high-performance monodisperse microcube scintillators made of copper iodide-(1-propyl-1,4-diazabicyclo[2.2.2]octan-1-ium)_2_ (Cu_4_I_6_(pr-ted)_2_) using a hot-injection method followed by thermal annealing at 200 °C for 1.5 h under a nitrogen atmosphere. Our experiments show that the as-prepared Cu_4_I_6_(pr-ted)_2_ microcubes exhibit strong green radioluminescence at 535 nm, which is identical to phosphorescence under 365 nm excitation, and enable a low X-ray detection limit of 22 nGy_air_ s^−1^. Additionally, we demonstrate that these microcube scintillators are remarkably robust to both water and X-rays. Moreover, we showcase the attractive applications of the uniform Cu_4_I_6_(pr-ted)_2_ microcubes as microfillers in the fabrication of flexible, high-performance composite scintillators for small animal X-ray imaging in both static and dynamic settings. Our results suggest that these microcubes hold great promise as advanced scintillator materials for radiation detection and imaging.

## Results

**Fig. 1:**
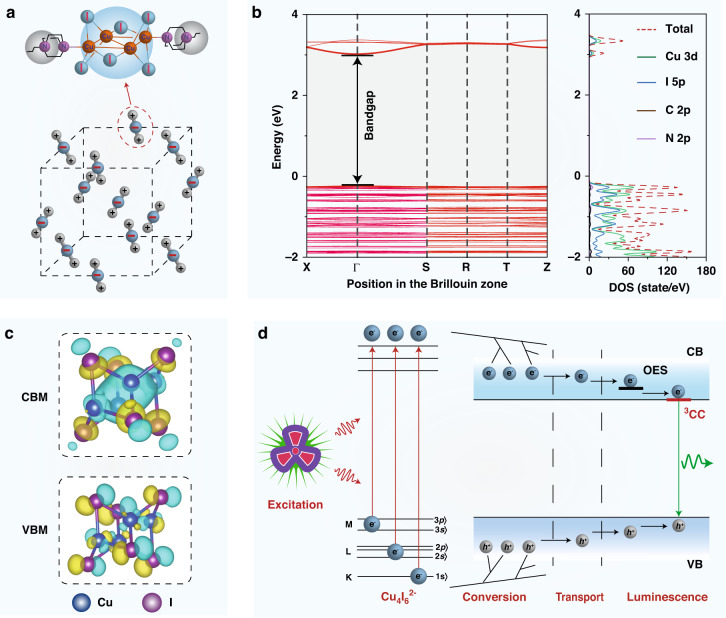
Construction of copper(I)-iodide cluster-based high-performance scintillators. **a** Schematic of the crystalline structure of the hybrid material Cu_4_I_6_(pr-ted)_2_. **b** The electronic band structure and density of states of Cu_4_I_6_(pr-ted)_2_. **c** The charge densities associated with the conduction band minimum and valence band maximum of Cu_4_I_6_(pr-ted)_2_. **d** The proposed mechanism for X-ray scintillation in Cu_4_I_6_(pr-ted)_2_ structures. Upon X-ray exposure, heavy elements such as Cu and I in the inorganic core strongly absorb high-energy photons, creating a large number of energetic primary electrons. This leads to the production of secondary electrons through a combination of photoelectric absorption, Compton scattering, and pair formation. As high-energy secondary electrons move within the host lattice, they lose energy through interactions with the lattice and other electrons, producing many excitons. These excitons are then transformed into low-energy scintillation photons through radiative recombination in the ^3^CC states of optical exciton states

To test our hypothesis, we selected Cu_4_I_6_(pr-ted)_2_) in the orthorhombic crystal structure P222 as our model (Fig. 1a)^37^. We used density functional theory to study the electronic band structure of the inorganic-organic hybrid Cu_4_I_6_(pr-ted)_2_ semiconductor. Our density functional theory (DFT) calculations showed a bandgap of 2.93 eV (Fig. 1b). The conduction band is mainly supported by Cu and I atomic orbitals, like Cu 3d and I 5p, as shown by the calculated conduction band minimum (CBM) and valence band maximum (VBM) (Fig. 1c). These results suggest that the Cu_4_I_6_^2-^ core has high electronic delocalization, which leads to efficient energy migration from the host to the emission centers and strong emission from triplet cluster-centered (^3^CC) excited states, as the excited energy converges from other excited states (OESs) (Supplementary Fig. S1)^38^. The combination of these intrinsic optical properties and the semiconducting nature of Cu_4_I_6_(pr-ted)_2_ crystals results in high scintillation performance when exposed to X-ray irradiation (Fig. 1d).

**Fig. 2:**
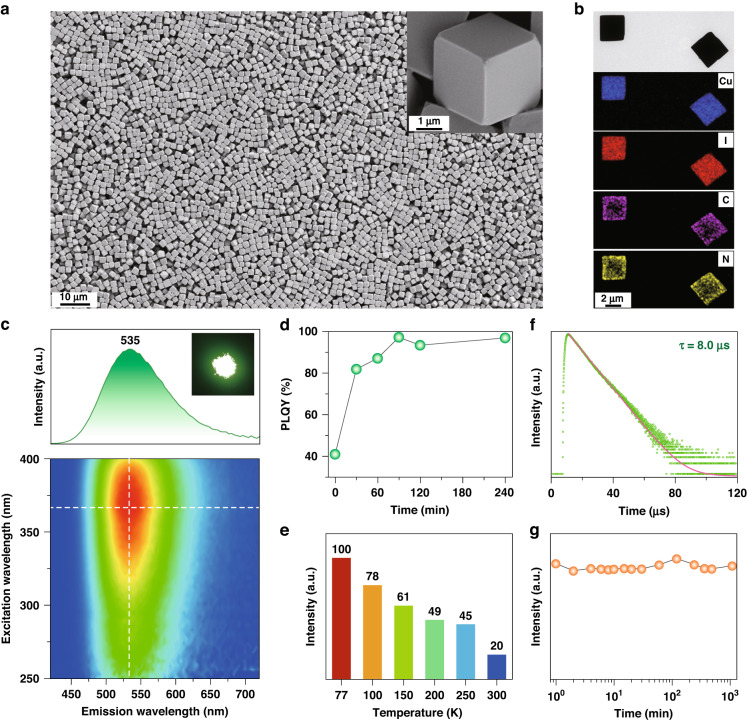
Characterization of Cu_4_I_6_(pr-ted)_2_ microcubes. **a** SEM image of the hot-injection prepared Cu_4_I_6_(pr-ted)_2_ microcubes. The inset shows a high-magnification SEM image of an individual microcube. **b** TEM and elemental mapping images of single microcubes. **c** Excitation-photoluminescence mapping of the Cu_4_I_6_(pr-ted)_2_ microcubes. The inset shows a photograph of the UV-induced luminescence of the microcubes (365 nm). **d** PLQYs of the microcubes as a function of annealing time (200 °C, N_2_ atmosphere). **e** Comparison of the photoluminescence intensity of the Cu_4_I_6_(pr-ted)_2_ microcubes measured at various temperatures. **f** Decay curve of the green emission (535 nm) of the microcubes. **g** Photoluminescence intensity of an aqueous dispersion of Cu_4_I_6_(pr-ted)_2_ microcubes as a function of incubation time

The preparation of Cu_4_I_6_(pr-ted)_2_ microcubes was carried out by injecting pr-ted into a mixture of KI-saturated CuI and polyvinylpyrrolidone (PVP) at 70 °C. The reaction was then quenched using a water-ice bath. X-ray diffraction (XRD) analysis confirmed the orthorhombic phase of the Cu_4_I_6_(pr-ted)_2_ microcubes, as supported by the match between the experimental and simulated XRD profiles (Supplementary Fig. S2). Scanning electron microscopy (SEM) revealed a uniform cubic morphology with an average size of 2.2 μm (Fig. 2a and Supplementary Fig. S3), with smooth and flat surfaces as observed under close inspection (Inset, Fig. 2a). Transmission electron microscopy also confirmed the cubic morphology of the Cu_4_I_6_(pr-ted)_2_ microcrystals (Fig. 2b), and elemental mapping showed a homogeneous distribution of Cu, I, C, and N elements in selected microcrystals. These results were consistent with X-ray photoelectron spectroscopy, which confirmed the presence of Cu, I, C, and N in the microcubes (Supplementary Fig. S4). The Cu component was identified as Cu^+^ based on the location of the Cu 2p_3/2_ peak at 929.1 eV^39^. The measured bandgap was 2.72 eV, which was slightly lower (0.21 eV) than the calculated value (Supplementary Fig. S5), likely due to delocalization error in the density functional approximations^40^.

Next, we found that the formation of uniform Cu_4_I_6_(pr-ted)_2_ microcubes requires precise control over nucleation kinetics. Traditional heating methods resulted in non-uniform spherical microparticles (Supplementary Fig. S6). Using room-temperature injection led to irregular Cu_4_I_6_(pr-ted)_2_ particles, indicating that slow nucleation kinetics hinders the formation of uniform microcubes (Supplementary Fig. S7). At room temperature, the Cu_4_I_6_(pr-ted)_2_ microcubes displayed a broad excitation band between 250–400 nm due to the inherent absorption of the building blocks^41^. Upon excitation at 370 nm, the microcubes showed maximum green emission at 535 nm (Fig. 2c) with a solid-state photoluminescence quantum yield (PLQY) of 40.7% (Supplementary Eq. (S4)). Annealing under a nitrogen atmosphere at 200 °C for different time periods (0.5, 1.0, and 1.5 h) increased the PLQY to 81.8%, 87.0%, and 97.1%, respectively (Fig. 2d). This annealing process significantly improved the crystallinity by reducing defect density in the Cu_4_I_6_(pr-ted)_2_ microcubes (Supplementary Fig. S2), while having minimal impact on their cubic shape and size distribution (Supplementary Fig. S8). Note that our DFT calculations suggest the large possibility of the presence of Cu interstitials other than I interstitials and Cu and I vacancies because of their low formation energy of 0.58 eV. Moreover, the high thermal stability of Cu_4_I_6_(pr-ted)_2_ microcubes in inert conditions was confirmed by the thermogravimetric analysis, which showed no apparent mass loss before 285 °C (Supplementary Fig. S9). These findings further support the attractiveness of Cu_4_I_6_(pr-ted)_2_ microcubes as high-performance optical materials that can be treated at high temperatures for improving their luminescence performance.

In a further set of experiments, we evaluated the temperature-dependent optical properties of the synthesized Cu_4_I_6_(pr-ted)_2_ microcubes. As the temperature decreased, we observed a narrowing of the emission profile, accompanied by a slight blue shift in the emission band (Supplementary Fig. S10). These observations were attributed to reduced structural torsion and an increased bandgap between the CBM and VBM of the Cu_4_I_6_(pr-ted)_2_ microcubes at low temperatures. Additionally, the green emission improved due to a decrease in temperature-induced dissipation of vibrational energy (Fig. 2e). The lifetime of the green emission was measured at 8.0 μs at room temperature (Supplementary Eq. (S5) and Fig. 2f) and was found to increase at low temperatures (Supplementary Fig. S11). These optical results confirm that the green emission originates from the ^3^CC excited states with phosphorescence^42^. Notably, the Cu_4_I_6_(pr-ted)_2_ microcubes showed remarkable resistance to degradation by water, as no noticeable changes in emission intensity were observed after 18 h of incubation in water (Fig. 2g). On the other hand, CsPbBr_3_, Cs_3_Cu_2_I_5_, and CsCu_2_I_3_ nano-/micro-particles showed high susceptibility to moisture, with complete degradation of CsPbBr_3_ and Cs_3_Cu_2_I_5_ occurring within a few minutes with just a small amount of water (Supplementary Figs. S12 and S13). A increased resistance of CsCu_2_I_3_ micro-rods may be due to a combination of their large size and low Cu^+^ solubility in the mixture (Supplementary Fig. S14)^43^. The improved water resistance of our Cu_4_I_6_(pr-ted)_2_ microcubes is likely to be achieved by introducing coordination bonding between Cu and I, and between Cu and organic ligand, as opposed to the ionic nature of conventional scintillation materials. These findings indicate that it is possible to synthesize uniform micro-sized crystalline structures with exceptional luminescence and structural robustness by utilizing Cu(I)-I cluster building blocks.

**Fig. 3:**
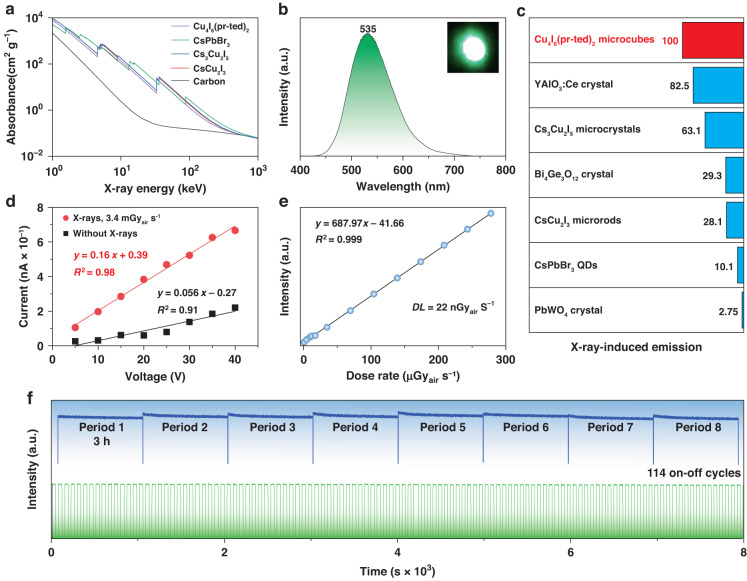
Radioluminescence characterization of Cu_4_I_6_(pr-ted)_2_ microcubes. **a** Comparison of the X-ray absorption profile of Cu_4_I_6_(pr-ted)_2_ and other reported scintillators. **b** Radioluminescence spectrum of Cu_4_I_6_(pr-ted)_2_ microcubes at an excitation dose of 278 μGy_air_ s^−1^. Inset: photograph of the radioluminescence image of the microcubes. **c** Radioluminescence comparison of Cu_4_I_6_(pr-ted)_2_ microcubes with other reported or commercially available scintillators under the same excitation conditions. **d** Photocurrent curve for a Cu_4_I_6_(pr-ted)_2_ microcube film with and without X-ray irradiation. Note that measurements were performed at different voltages. **e** Radioluminescence intensity of Cu_4_I_6_(pr-ted)_2_ microcubes as a function of excitation dose. Note that a *DL* of 22 nGy_air_ s^−1^ is obtained via the 3σ/slope method. **f** X-ray tolerance of Cu_4_I_6_(pr-ted)_2_ microcubes against continuous X-ray irradiation (dose rate: 2.85 mGy_air_ s^−1^; top) and repeated X-ray irradiation (dose rate: 1.39 mGy_air_ s^−1^, with an interval of 30 s, bottom). Note that the radioluminescence at 535 nm was monitored to check the robustness of Cu_4_I_6_(pr-ted)_2_ microcubes

The solid-state radioluminescence of Cu_4_I_6_(pr-ted)_2_ microcubes was investigated at room temperature. The calculated effective atomic number of Cu_4_I_6_(pr-ted)_2_ was 45.6, which is comparable to the X-ray absorption abilities of other materials such as CsPbBr_3_, Cs_3_Cu_2_I_5_, and CsCu_2_I_3_ (Fig. 3a and Supplementary Table S1)^44–46^. The Cu_4_I_6_(pr-ted)_2_ microcubes exhibited strong single-band green radioluminescence at 535 nm upon X-ray excitation at a dose rate of 278 μGy_air_ s^−1^ (Fig. 3b and inset). The observed radioluminescence was found to be consistent with the photoluminescence, suggesting a common origin from the ^3^CC excited states. These findings affirm the mechanism responsible for the strong radioluminescence displayed in Fig. 1d. The high-energy X-rays used in the experiment led to the ejection of numerous energetic primary electrons from the inner shells of both Cu and I atoms, along with the creation of holes. These primary electrons generated secondary electrons through kinetic energy dissipation during transport, resulting in the formation of a significant number of excitons in various excited states. These excitons were then converted to low-energy ^3^CC excited states, which produced efficient radioluminescence through radiative recombination.

The sensitivity of Cu_4_I_6_(pr-ted)_2_ microcubes to X-ray excitation was quantitatively compared with several commercially available or reported scintillators using the same dose rate of X-rays (Fig. 3c, Supplementary Fig. S15, and Supplementary Table S2). We also fixed the size of our scintillation film to match that of conventionally available scintillation single crystals, with both having the same diameter (1.0 cm) and thickness (1.0 mm). Results showed that a thin film of the Cu_4_I_6_(pr-ted)_2_ microcubes delivered radioluminescence that was 1.21, 3.41, and 36.36 times stronger than a single crystal of YAlO_3_:Ce, Bi_4_Ge_3_O_12_, and PbWO_4_, respectively. In comparison to corresponding micro-/nano-particle films of Cs_3_Cu_2_I_5_, CsCu_2_I_3_, and CsPbBr_3_ under the same excitation conditions, the radioluminescence of Cu_4_I_6_(pr-ted)_2_ microcubes was also 1.58, 3.56, and 9.90 times stronger, respectively. These observations support the hypothesis that Cu_4_I_6_(pr-ted)_2_ microcubes exhibit not only remarkable X-ray stopping power but also high energy conversion performance. The conductivity of a film of Cu_4_I_6_(pr-ted)_2_ microcubes in the dark was also considerable, indicating their semiconducting nature and the feasibility of high-efficiency energy migration from the host to the emission centers, resulting in strong scintillation emissions (Fig. 3d and Supplementary Fig. S16). The photoconductive gain testing curve showed a higher current output upon X-ray irradiation at a dose rate of 3.4 mGy_air_ s^−1^, suggesting the conversion of X-rays to visible photons in Cu_4_I_6_(pr-ted)_2_ microcubes occurs through the formation of X-ray-induced charge carriers. As a result, Cu_4_I_6_(pr-ted)_2_ microcubes exhibited a low detection limit (*DL*) of 22 nGy_air_ s^−1^ (Fig. 3e), which is approximately 250 times lower than the standard dose for medical X-ray examinations (5.5 μGy_air_ s^−1^)^47^. Additionally, the high robustness of Cu_4_I_6_(pr-ted)_2_ microcubes to X-ray irradiation was demonstrated, as no noticeable degradation was observed during both continuous irradiations for 3 h and 8 cycles (2.85 mGy_air_ s^−1^) and repeated X-ray irradiation at 30-second intervals for 114 on-off cycles (1.39 mGy_air_ s^−1^).

The strong radioluminescence emission of Cu_4_I_6_(pr-ted)_2_ microcubes in response to X-rays allows for the development of a flexible energy-conversion substrate for X-ray imaging. A large-sized polydimethylsiloxane (PDMS) film (169 cm^2^, 0.5 mm thick) was fabricated by doping with 5.0 wt% of Cu_4_I_6_(pr-ted)_2_ microcubes. The doped PDMS film demonstrated excellent flexibility, significant transparency, and emitted homogeneous and intense green radioluminescence upon X-ray excitation (Supplementary Fig. S17). These results suggest that the Cu_4_I_6_(pr-ted)_2_ microcubes maintain their exceptional scintillation properties even after doping into the PDMS film. In addition, we found that doping Cu_4_I_6_(pr-ted)_2_ microcubes at 5 wt% slightly increased the mechanical properties of the PDMS film, as evidenced by an increase in Young’s modulus from 0.37 to 0.78 MPs (Supplementary Fig. S18). The enhanced mechanical strength can be attributed to the homogeneous distribution of the microcubes that have strong interactions with the PDMS matrix.

**Fig. 4:**
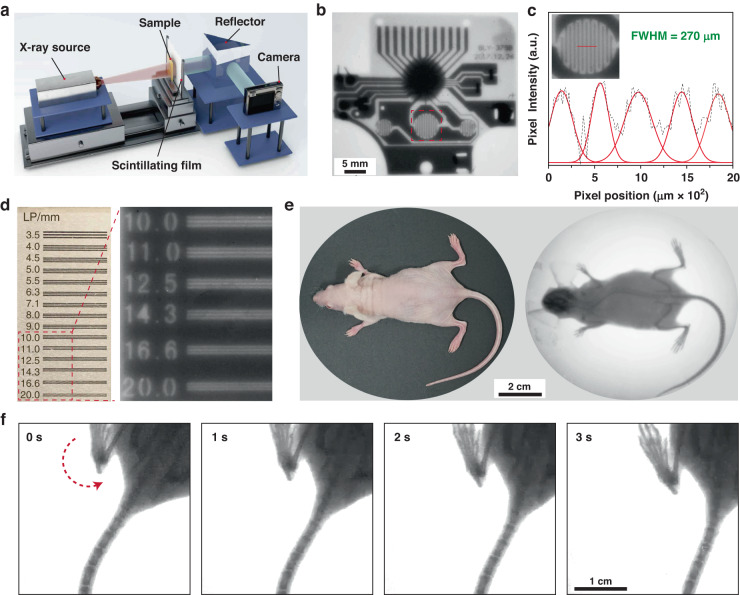
Static and dynamic X-ray imaging of a flexible PDMS film doped with Cu_4_I_6_(pr-ted)_2_ microcubes. **a** Schematic representation of the X-ray imaging setup, which includes a X-ray source, a flexible scintillating screen, a reflector and a digital camera. **b** X-ray image of a printed circuit board demonstrating the imaging capabilities. **c** Evaluation of the spatial resolution through the analysis of the intensity spread profile of the circuit pattern, as shown in the inset. **d** X-ray imaging of a standard X-ray pattern plate with a resolution range of 11–20 lp mm^−1^. **e** Comparison of bright-field and X-ray images of a mouse. **f** Dynamic X-ray imaging of the mouse without ghosting at a rate of 6.45 frames per second

X-ray imaging was performed using a homemade setup (Fig. 4a) and resulted in a clear visualization of the detailed structure of a timer-printed circuit board (Fig. 4b and Supplementary Fig. S19). The imaging produced a full width at half maximum (FWHM) of 270 μm (Fig. 4c) and a resolution of 20.00 line pairs per millimeter (Fig. 4d). Conversely, using irregular Cu_4_I_6_(pr-ted)_2_ microparticles as scintillating dopants resulted in aggregates in the plastic film surface, leading to defects in the X-ray images (Supplementary Fig. S20). Note that the resolution decreased to 3.5 lp/mm when increasing the thickness of the scintillating film to 1.0 mm as a result of an increased scattering effect (Supplementary Fig. S21). In addition, we found that the X-ray imaging performance of our scintillation film did not deteriorate even after 2000 cycles of cyclic bending, suggesting that our scintillation film has a high anti-fatigue property (Supplementary Fig. S22). X-ray imaging of a nude mouse was also performed using the Cu_4_I_6_(pr-ted)_2_ microcube-doped flexible screen and produced a clear image of the entire mouse skeleton (Fig. 4e), especially the tailbone where spacings between individual bones are clearly visible at about 226 μm (Supplementary Fig. S23). The radioluminescence of the as-prepared Cu_4_I_6_(pr-ted)_2_ microcubes rapidly decreases to background levels (within 10 ms) after cessation of X-ray excitation (Supplementary Fig. S24), making the doped PDMS film particularly suitable for real-time dynamic X-ray imaging with no ghosting effect observed at an imaging rate of 6.45 frames per second (Fig. 4f, Supplementary Fig. S25, and Supplementary Movie S1)^48^.

## Discussion

In this study, we report on the potential of Cu(I)-I cluster structures as a new class of highly stable and eco-friendly scintillators due to their composition and optical characteristics. The high scintillation performance of the as-prepared Cu_4_I_6_(pr-ted)_2_ microcubes allows for a low X-ray detection limit of 22 nGy_air_ s^−1^. With the abundant choice of inorganic cores available, the X-ray absorption capability of Cu(I)-I cluster crystals can be further improved by introducing more I atoms while maintaining the high efficiency of converting X-rays into low-energy photons. Moreover, modulating the organic ligand shows promise in tuning the excited-state transitions in Cu(I)-I cluster crystals, enabling feasible color tuning of radioluminescence in the visible region.

A particularly attractive aspect of our work is that monodisperse Cu_4_I_6_(pr-ted)_2_ microcubes can be produced using a kinetically controlled synthesis method, making them ideal for use as micro-sized scintillators in the development of large-scale and flexible scintillation screens for both static and dynamic X-ray imaging. Compared with conventional metal halide scintillation materials, Cu_4_I_6_(pr-ted)_2_ microcubes exhibit excellent chemical stability. This feature not only provides considerable benefits in practical device manufacturing but also offers the potential to regulate the morphology and dimensions of Cu(I)-I cluster nanoscintillators to enhance their scintillation performance. For example, doping Cu_4_I_6_(pr-ted)_2_ microcubes into a polymer matrix allows for the fabrication of plastic scintillating screens for flexible X-ray detection and imaging.

Overall, our results indicate that Cu_4_I_6_(pr-ted)_2_ microcubes possess significant practical advantages, including enhanced chemical and optical stability, waterproofing, and low toxicity. These findings should stimulate further study on Cu(I)-I cluster materials to explore next-generation scintillators for innovative radiography.

## Methods

### Synthesis of pr-ted

The synthesis of pr-ted was conducted using a modified version of a previously reported method^41^. To synthesize pr-ted, 1-bromopropane (10 mmol) was dropped into a 50 mL acetone solution of triethylenediamine (Ted, 0.2 M) under magnetic stirring, forming a clear solution. This solution was then left at room temperature for 12 h, yielding a colorless oily product. The product was recovered by centrifugation, washed with ethyl acetate, and dried under vacuum. The final yield was estimated to be 68 wt%.

### Synthesis of Cu_4_I_6_(pr-ted)_2_ microcubes

In a typical experiment, PVP (K88-96, 0.02 g/mL, 12.5 mL) was dissolved in ethanol to form a solution, and a KI-saturated aqueous solution of CuI (0.5 mmol mL^−1^, 0.5 mL) was prepared. The two solutions were then mixed in a 50-mL flask at 70 °C while stirring with a magnet, resulting in a homogeneous pale yellow solution. An ethanol solution of pr-ted (0.5 mmol mL^−1^, 0.5 mL) was then injected to the CuI-KI-PVP solution at 70 °C, and the mixture was immediately immersed in a water-ice bath. The final product, Cu_4_I_6_(pr-ted)_2_ microcubes, was obtained through centrifugation at 6000 rpm for 5 min, followed by washing twice with deionized water and ethanol. The purified Cu_4_I_6_(pr-ted)_2_ microcubes were then treated at 200 °C in a nitrogen atmosphere for different periods of time.

### Synthesis of the flexible PDMS film doped with Cu_4_I_6_(pr-ted)_2_ microcubes

In brief, treated Cu_4_I_6_(pr-ted)_2_ microcubes (0.396 g) were mixed with a 1:1 solution of cyclohexane and ethanol (10 mL) under sonication to form a homogeneous dispersion. This dispersion was then combined with a mixture of PDMS prepolymer (7.2 g) and curing agent (0.72 g) under vigorous stirring for 2 h. The mixture was then subjected to vacuum for 1 h to remove any volatile materials. The resulting gel-like substance was poured into a polytetrafluoroethylene mold (13 × 13 cm^2^) and cured at 200 °C for 1.5 h. The result was a flexible and transparent PDMS film doped with Cu_4_I_6_(pr-ted)_2_ microcubes suitable for imaging applications.

### Instrumentation

Scanning electron microscopy was performed using a Zeiss Gemini 300 microscope at a voltage of 3 kV. Transmission electron microscopy was conducted on a Hitachi HT 7700 operating at 120 kV. Powder X-ray diffraction characterization was carried out using a Bruker D8 Advance X-ray diffractometer with Cu Kα radiation. Photoluminescence emission profiles and decay curves were obtained using an FSL-1000 (Edinburgh Instruments Ltd.). PLQY measurements were performed on a C9920-02G system (Hamamatsu). Radioluminescence emission profiles were acquired using an Edinburgh FS5 fluorescence spectrophotometer (Edinburgh Instruments Ltd.), equipped with an external miniature X-ray source from AMPEK, Inc.

## Supplementary information

Supplementary Information for Efficient X-ray luminescence imaging with ultrastable and eco-friendly copper(I)-iodide cluster microcubes

Dynamic X-ray imaging study
