# Supplementary material for: Efficient X-ray luminescence imaging with ultrastable and eco-friendly copper(I)-iodide cluster microcubes
Source: Light Sci Appl. 2023 Jun 25;12:155. doi: 10.1038/s41377-023-01208-0 (PMC10290987; doi:10.1038/s41377-023-01208-0)
Supplement: Supplementary file 1 — Supplementary Information for Efficient X-ray luminescence imaging with ultrastable and eco-friendly copper(I)-iodide cluster microcubes [file 41377_2023_1208_MOESM1_ESM.docx]

**Supplementary Information for**

**Efficient X-ray luminescence imaging with ultrastable and eco-friendly copper(I)-iodide cluster microcubes**

Yanze Wang^1^, Wenjing Zhao^1^, Yuanyuan Guo^1^, Wenbo Hu^1^, Chenxi Peng^1^, Lei Li^1,2^, Yuan Wei^1^, Zhongbin Wu^1^, Weidong Xu^1^, Xiyan Li^3^, Yung Doug Suh^4^, Xiaowang Liu^1^*, and Wei Huang^1,5,6^*

^1^Frontiers Science Center for Flexible Electronics (FSCFE), MIIT Key Laboratory of Flexible Electronics (KLoFE), Shaanxi Key Laboratory of Flexible Electronics, Xi'an Key Laboratory of Flexible Electronics, Xi'an Key Laboratory of Biomedical Materials & Engineering, Xi'an Institute of Flexible Electronics, Institute of Flexible Electronics (IFE), Northwestern Polytechnical University, Xi'an 710072, Shaanxi, China.

^2^Key Laboratory of Magnetic Materials Devices, Ningbo Institute of Materials Technology and Engineering, Chinese Academy of Sciences, Ningbo 315201, China.

^3^Institute of Photoelectronic Thin Film Devices and Technology, Solar Energy Conversion Center, Nankai University, Tianjin 300350, China.

^4^Department of Chemistry and School of Energy and Chemical Engineering, UNIST, Ulsan 44919, Korea.

^5^State Key Laboratory of Organic Electronics and Information Displays & Institute of Advanced Materials(IAM), Nanjing University of Posts & Telecommunications, 9 Wenyuan Road, Nanjing 210023, China.

^6^Key Laboratory of Flexible Electronics (KLOFE), Institute of Advanced Materials (IAM), Nanjing Tech University (Nanjing Tech), 30 South Puzhu Road, Nanjing 211816, China.

Content

I. Experimental Section S2

II. Understanding the photoluminescence of copper(I)-iodide cluster structure S3

III. Evaluation of X-ray attenuation coefficient, effective atomic number and light yield S3

IV Physical Measurement.. S3

V. DFT Calculations S4

VI. Chemical Stability Examination S5

VII. Static and dynamic X-ray imaging S5

VIII. Supplementary Figures S6

IX. Supplementary Tables S31
X. References S33

**I. Experimental Section**

**Chemicals.** All chemicals were utilized as received, including cuprous iodide (CuI, 99.999%, Alfa Aesar), potassium iodide (KI, 99%, Alfa Aesar), polyvinylpyrrolidone (PVP, K88-96, molecular weight of 1300000, Innochem Technology Co., Ltd), ethanol (99.7%, Shanghai Titan Scientific Co., Ltd), triethylenediamine (Ted, 98%, Alfa Aesar), 1-bromopropane (99%, Alfa Aesar), acetone (≥ 99.5%, Shanghai Titan Scientific Co., Ltd), ethyl acetate (99.7%, Shanghai Titan Scientific Co., Ltd), cesium carbonate (Cs_2_CO_3_, 99.99%, Aladdin), lead(II) bromide (PbBr_2_, 99.99%, Innochem Technology Co., Ltd), cyclohexane (≥ 99.5%, Shanghai Titan Scientific Co., Ltd), oleylamine (80-90%, Aladdin), oleic acid (90%, from Alfa Aesar), and octadecene (90%, Alfa Aesar). The single crystal scintillators of YAlO_3_:Ce, Bi_4_Ge_3_O_12_, and PbWO_4_ (with a thickness of 1 mm) were purchased from Zhonghelixin Co., Ltd (Chengdu, China).

**Synthesis of CsPbBr_3_ NPs.** CsPbBr_3_ NPs were synthesized using a modified version of a previous method^1^. To start, Cs-oleate was prepared by heating Cs_2_CO_3_ (0.615 mmol), oleic acid (0.625 mL), and octadecene (7.5 mL) at 100 °C under vacuum for 0.5 h to remove volatile impurities. The reaction was then stirred at 150 °C until the mixture became pale yellow and transparent. After cooling to 100 °C, a Pb^2+^ precursor solution was prepared by adding PbBr_2_ (0.188 mmol) to a mixture of oleic acid (0.5 mL), oleylamine (0.5 mL), and octadecene (5 mL) in a 25-mL flask. This mixture was heated at 100 °C for 0.5 h to remove impurities and then at 160 °C under a nitrogen atmosphere to dissolve PbBr_2_. The hot Cs-oleate precursor solution (0.5 mL) was then injected into the Pb^2+^ precursor solution and heated for another 5 s before being quickly cooled in an ice bath. The CsPbBr3 NPs were obtained by centrifugation at 6000 rpm for 10 minutes, washed with a 1:1 mixture of cyclohexane and ethanol, and stored in 3 mL of cyclohexane for further use.

**Synthesis of Cs_3_Cu_2_I_5_ microcrystals.** Cs_3_Cu_2_I_5_ microcrystals were synthesized using a previously reported method with slight modifications^2^. The process began with the preparation of Cs-oleate by heating Cs_2_CO_3_ (0.615 mmol), oleic acid (0.625 mL), and octadecene (7.5 mL) at 100 °C under vacuum for 0.5 h to remove volatile impurities. The temperature was then increased to 150 °C under vigorous stirring until the mixture became pale yellow and transparent. After removing the heating source, the Cs-oleate was allowed to cool to 100 °C. Next, a Cu^+^ precursor solution was prepared by heating a mixture of CuI (0.2 mmol), oleic acid (0.5 mL), oleylamine (0.5 mL), and octadecene (5 mL) at 120 °C for 0.5 h to remove volatile impurities. The mixture was then heated to 150 °C under an N_2_ atmosphere to ensure the complete dissolution of the CuI powder. The hot Cs-oleate precursor solution (0.5 mL) was then rapidly injected into the Cu^+^ precursor solution at 150 °C. After heating for an additional 30 s, the flask was transferred to an ice bath for cooling. The Cs_3_Cu_2_I_5_ microcrystals were collected by centrifugation at 6000 rpm for 10 min, washed with a mixture of cyclohexane and ethyl acetate (v/v: 1:4), and stored in cyclohexane (3 mL) for future use.

**Synthesis of CsCu_2_I_3_ microcrystals.** CsCu_2_I_3_ microcrystals were synthesized based on a previously reported method with slight modifications^3^. In a typical experiment, Cs-oleate was first prepared by heating Cs_2_CO_3_ (0.468 mmol), oleic acid (0.475 mL), and octadecene (7.5 mL) at 100 °C under vacuum for 0.5 h to remove volatile impurities. The reaction temperature was then increased to 150 °C under vigorous stirring until the mixture became pale yellow and transparent. The heating source was then removed, and the as-prepared Cs-oleate was allowed to cool to 100 °C. A Cu^+^ precursor solution was then prepared by heating a mixture of CuI (0.2 mmol), oleic acid (0.5 mL), oleylamine (0.5 mL), and octadecene (5.0 mL) at 120 °C for 0.5 h under vacuum to remove volatile impurities. The mixture was then heated at 150 °C under an N_2_ atmosphere to completely dissolve the CuI powder. A hot Cesium-oleate precursor solution (1.5 mL) was then injected into the resultant Cu^+^ precursor solution and heated for another 30 s. The flask was then transferred to an ice bath until the temperature cooled to room temperature. The CsCu_2_I_3_ microcrystals were harvested by centrifugation at 6000 rpm for 10 min, washed with a mixture of cyclohexane and ethyl acetate (v/v: 1/4), and stored in cyclohexane (3 mL) for further use.

**II. Understanding the photoluminescence of copper(I)-iodide cluster structure**

Copper(I)-iodide cluster structures possess exceptional optical properties due to their multiple electronic transitions, such as intercore *d*→*s*,*p* metal-centered transitions (^3^CC, the triplet cluster-center state), metal-to-ligand charge transfer (^n^MLCT), counterion-to-ligand charge transfer (^n^XLCT), ligand local excited state (^n^LLE), and intraligand charge transfer (^n^ILCT). The *n* represents either 1 or 3, indicating singlet and triplet states, respectively. The ^3^CC state, with the lowest energy position, merges the excited energy from other states (^n^(M+X)LCT, ^n^LLE, and ^n^ILCT) through energy migration (Figure S1)^4,5^, leading to intense emission with phosphorescence.

**III.** **Evaluation of X-ray attenuation coefficient, effective atomic number, and light yield.**

The X-ray attenuation coefficient (*μ*) of a scintillating material can be estimated using Equation S1^6^.

$\mu=\frac{\rho Z^{4}}{AE^{3}}$ (1)

where *ρ* represents density, *Z* represents an atomic number, *A* represents atomic mass, and *E* represents incident X-ray energy. Note that for compounds, the effective atomic number (*Z_eff_*) is used, which can be estimated using Equation S2^7^.

 (2)

where *w_i_* is the weight ratio of the *i*-th element of the scintillating material, and *Z_i_* is the atomic number (*Z*) of the *i*-th element. The obtained effective atomic numbers for commercially available and reported scintillating materials were listed in Table S1.

The light yield can be evaluated by Equation S3^7^.

$I\text{ }=\text{ }\frac{E}{\beta E_{g}}*S*Q$ (3)

Where *E* is the energy of the incident X-rays, *β* is the constant parameter, *E_g_* is the bandgap energy, *S* is the host-to-emission center energy migration efficiency, and *Q* is the quantum efficiency, which is equivalent in value to the PLQY.

**IV. Physical Measurement**

**Instrumentation.** Scanning electron microscopy was performed using a Zeiss Gemini 300 microscope at a voltage of 3 kV. Transmission electron microscopy was conducted on a Hitachi HT 7700 operating at 120 kV. Powder X-ray diffraction characterization was carried out using a Bruker D8 Advance X-ray diffractometer with Cu Kα radiation. Photoluminescence emission profiles and decay curves were obtained using an FSL-1000 (Edinburgh Instruments Ltd.). PLQY measurements were performed on a C9920-02G system (Hamamatsu). Radioluminescence emission profiles were acquired using an Edinburgh FS5 fluorescence spectrophotometer (Edinburgh Instruments Ltd.), equipped with an external miniature X-ray source from AMPEK, Inc.

**PLQY measurement.** PLQY measurement was conducted on a C9920-02G absolute quantum yield measurement system (Hamamatsu Photonics). The Cu_4_I_6_(pr-ted)_2_ microcubes were placed in a quartz sample holder, and the emission was directed to the detector via an integrating sphere. The PLQY magnitude was estimated using Equation S4^8^.

 (4)

where *N_emitted_* and *N_absorbed_* represent the numbers of photons emitted and absorbed, respectively.

**Lifetime measurement.** In brief, the green emission lifetime of Cu_4_I_6_(pr-ted)_2_ microcubes was estimated using time-resolved photoluminescence spectroscopy. The single-exponential fitting method was applied to examine the radiative decay according to Equation S5^8^.

 (5)

where *I*(*t*) stands for the emission intensity at time *t*, and *I_0_* and *τ* represent the initial emission intensity and lifetime, respectively.

**Radioluminescence and detection limit measurement.** In brief, the radioluminescence and detection limit were measured using an Edinburgh FS5 fluorescence spectrophotometer, which was equipped with a commercially available miniature X-ray tube. The distance between the X-ray source and the Cu_4_I_6_(pr-ted)_2_ microcubes was fixed at 1.5 cm, and the radioluminescence was measured at a dose rate of 278 μGy_air_ s^-1^. The detection limit was calculated using a combination of the slope of the fitting line and the 3σ/slope method.

**X-ray photoconductor devices.** In brief, 50-nm-thick gold electrodes were deposited onto a silica wafer with a 300-nm-thick SiO_2_ layer using thermal evaporation and a shadow mask to control the size of the deposition. The X-ray photon-to-current measurement was performed using a Keithley 2450 digital source meter and a commercially available external miniature X-ray source. The X-ray source voltage was set at 50 kV, and the output was fixed at 4 W, with a 2-mm-diameter brass collimator. The distance between the X-ray source and the X-ray photoconductive device was 1 cm.

**V. DFT Calculations.**

Our DFT calculations were carried out using the Vienna ab initio simulation package and the generalized gradient approximation functional^9-11^. To ensure accuracy, we used a hybrid density functional of Heyd-Scuseria-Ernzerhof1^12,13^. The system being analyzed consisted of molecules, so we employed a uniform Monkhorst-Pack K mesh of 1x1x1 for integration over the Brillouin zone.

The formation energy of a vacancy (*E_f_*) was calculated using Equation S6.

$E_{f}=E_{v}-E_{0}+E_{i}$ (6)

where *E_v_* and *E_0_* are the total energy of the vacancy-containing and initial configurations, respectively. *E_i_* is the energy of the atom *i* removed from the initial crystal structure that generate a vacancy. During calculation, The calculated formation energies of the copper, iodide and nitrogen vacancies are 2.95, 2.75 and 2.45 eV respectively

The formation energy of an interstitial was calculated using Equation S7.

$E_{f}=E_{\mathrm{int}er}-E_{0}-E_{i}$ (7)

where *E_inter_* is the total energy of the interstitial-containing. The calculated formation energies are 0.58 and 1.11 eV for Cu and I interstitials respectively.

**VI. Chemical Stability Examination**

**Examination of resistance of Cu_4_I_6_(pr-ted)_2_ microcubes towards water degradation.** Typically, the as-prepared Cu_4_I_6_(pr-ted)_2_ microcubes were dispersed in de-ionized water (13.4 mg mL^-1^, 3 mL) to form a homogeneous mixture. The emission profiles were measured at various time intervals. The mixture was sonicated before each measurement to avoid the formation of precipitate at the bottom of the cuvette. The emission intensity was used to quantitatively evaluate the stability of Cu_4_I_6_(pr-ted)_2_ microcubes in water.

**Examination of resistance of nano-/micro-sized scintillators towards water degradation.** In a typical experiment, 42.8 mg of as-prepared nano-/micro-scintillators including CsPbBr_3_, Cs_2_Cu_2_I_5_, and CsCu_2_I_3_ were dispersed in a mixture of cyclohexane (1.5 mL) to form homogeneous dispersions. Then, a mixture of ethanol and water (v/v: 15/1, 1.6 mL) was added to the corresponding dispersion, and the emission profiles were acquired at various time intervals.

**VII. Static and dynamic X-ray imaging.**

**Static X-ray imaging.** Static X-ray imaging was performed using a homemade setup as depicted in Fig. 4a. Unless otherwise stated, the parameters were fixed, with a voltage of 70 kV, an anode material of gold, and a focal spot size of approximately 2 mm. In a typical experiment, a timer-printed circuit board was placed between an external miniature X-ray source and a Cu_4_I_6_(pr-ted)_2_ microcube-doped PDMS film. The X-ray source was positioned approximately 5 cm away from the timer circuit board.

**Dynamic X-ray imaging.** In a typical experiment, dynamic X-ray imaging was carried out using a customized 5x5-inch dynamic flat-panel detector (NDT 0505J) supplied by iRAY Technology Shanghai, Inc. The active area of the photodiode array is 13.0 x 13.0 cm^2^ and the α-Si TFT sensor has an 85 µm pixel pitch. In this experiment, the flexible PDMS film doped with Cu_4_I_6_(pr-ted)_2_ microcubes was used to replace the scintillating material layer in the dynamic flat-panel detector. The X-rays were supplied by an external miniature X-ray source operating at 70 kV.

**VIII. Supplementary Figures**

**
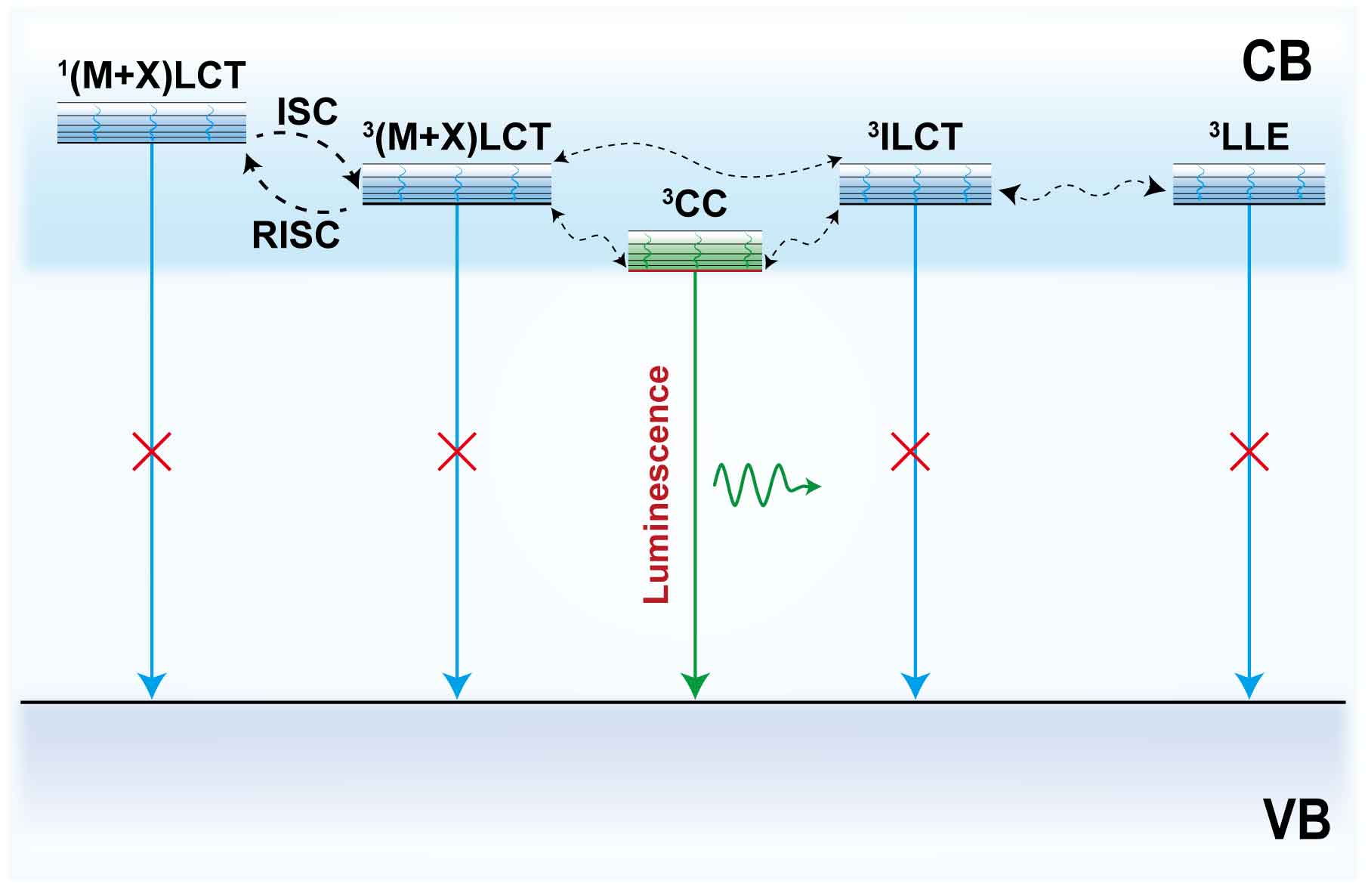
**

**Figure S1. Schematic of the deactivation of the excitation energy and energy transfer process in copper(I)-iodide cluster structures**. Typical excited-state transitions include mixed metal and counterion-ligand charge transfer transition (^n^(M+X)LCT), intraligand charge transfer (^n^ILCT), and ligand local excited transition (^n^LLE) (*n* = 1 for singlet and 3 for triplet states). ISC and RISC represent intersystem crossing and reverse intersystem crossing, respectively.

**
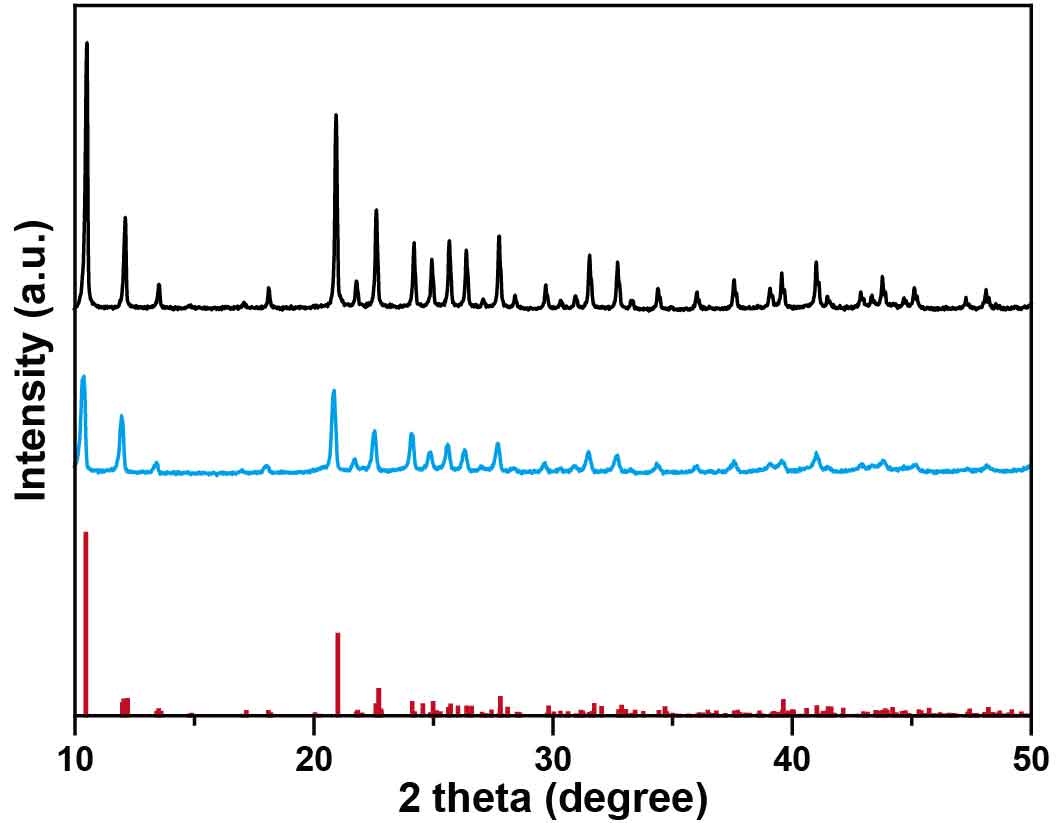
**

**Figure S2. XRD characterization.** XRD patterns of Cu_4_I_6_(pr-ted)_2_ microcubes before (blue) and after (black) being calcined at 200 °C for 1.5 h. The simulated XRD profile is presented in red.

**
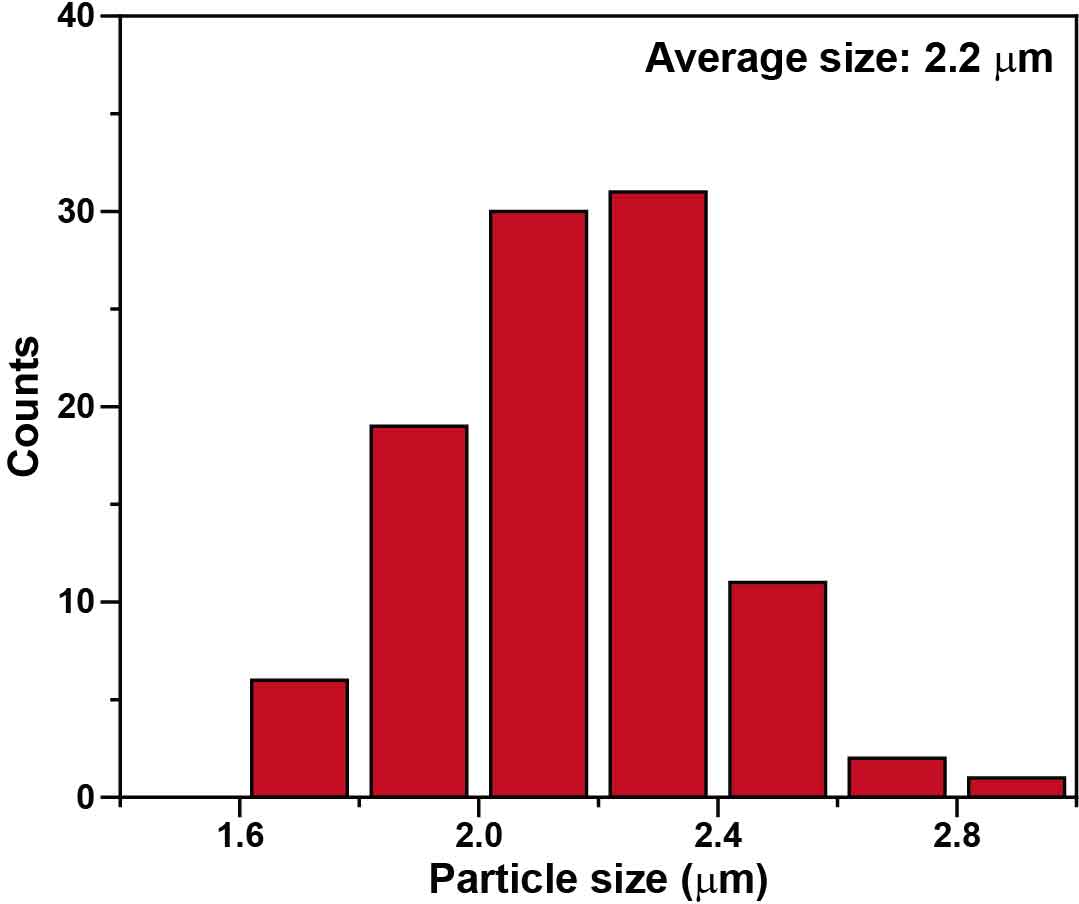
**

**Figure S3. The size distribution of Cu_4_I_6_(pr-ted)_2_ microcubes.** Note that 100 microcubes were used for the analysis.

**
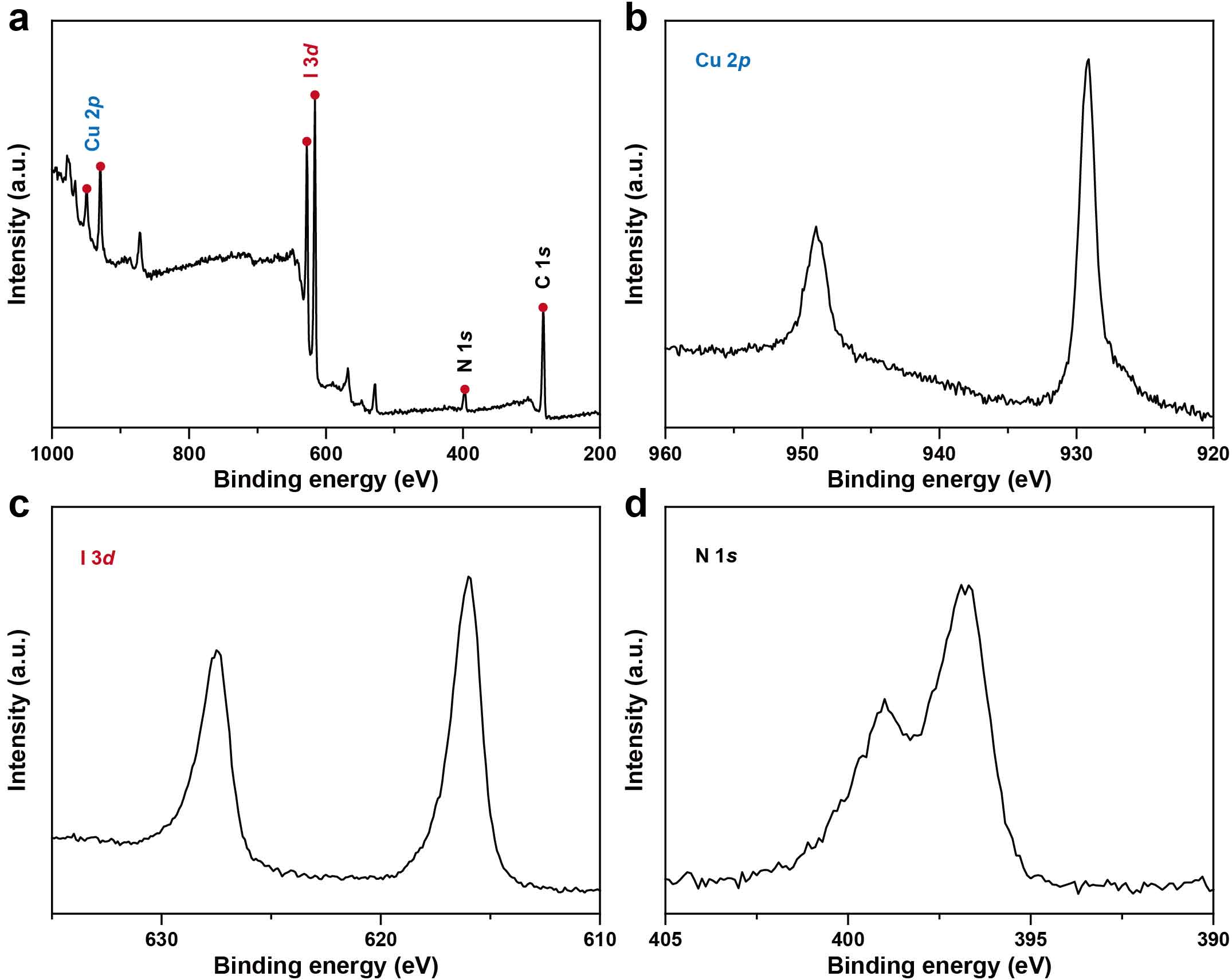
**

**Figure S4. XPS characterization. (a)** XPS of Cu_4_I_6_(pr-ted)_2_ microcubes. **(b, c, and d)** High-resolution XPS spectra of Cu 2*p*, I 3*d*, and N 1*s*.


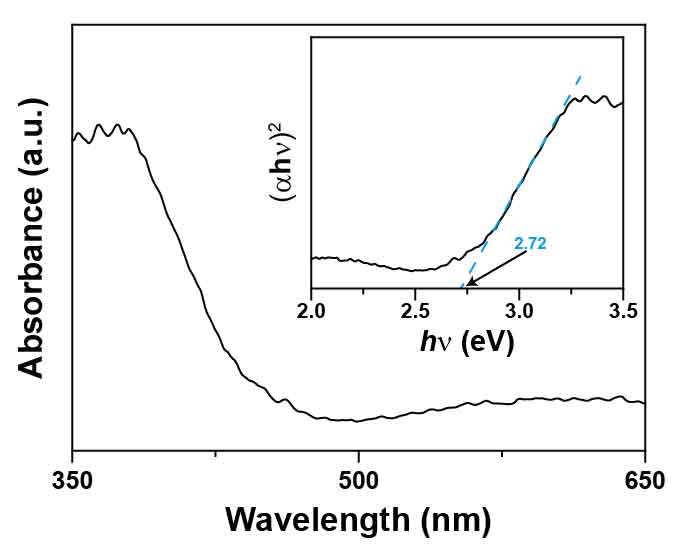


**Figure S5. UV-vis absorption spectrum of Cu_4_I_6_(pr-ted)_2_ microcubes.** Based on the measured curve, a bandgap of 2.72 eV was obtained for the Cu_4_I_6_(pr-ted)_2_ microcubes.

**
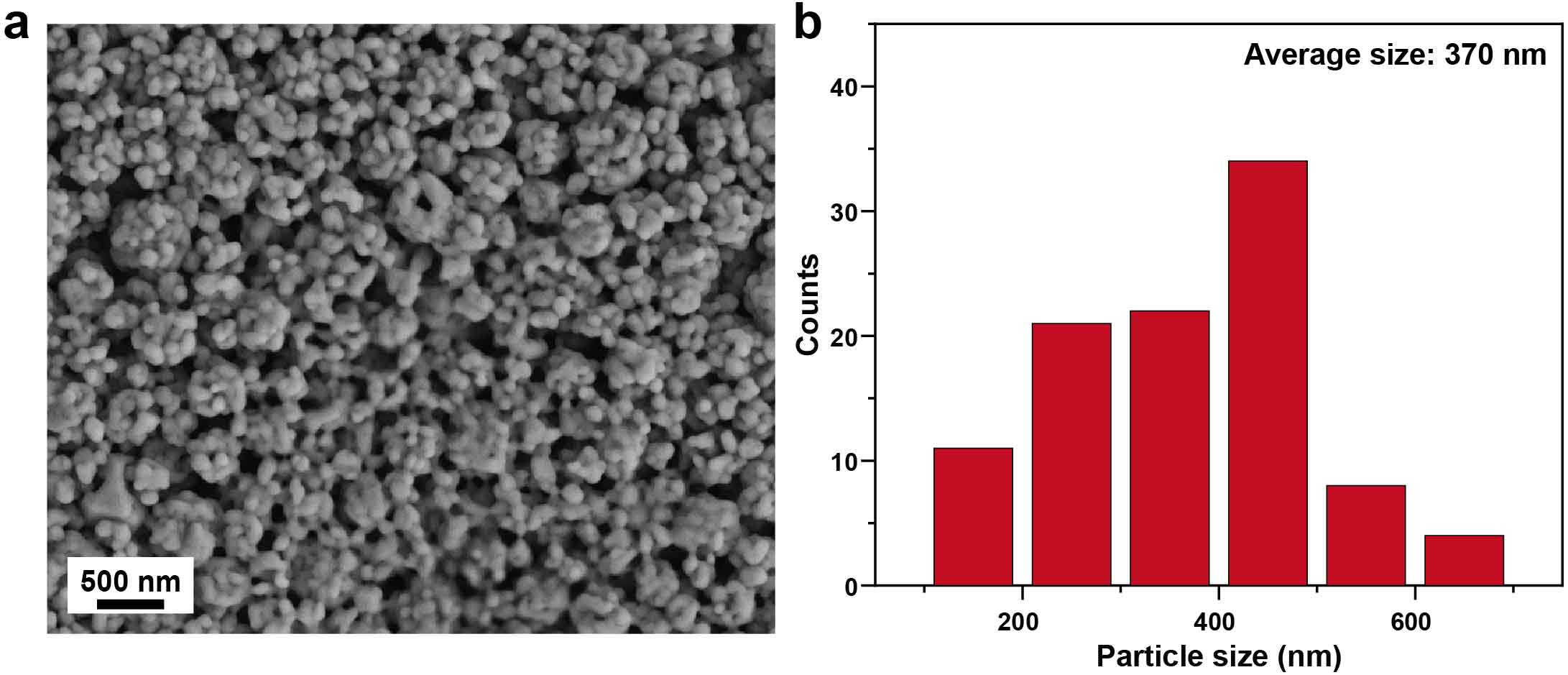
**

**Figure S6. Characterization of Cu_4_I_6_(pr-ted)_2_ microparticles obtained through a conventional heating method. (a)** SEM image and **(b)** size distribution graph.

**
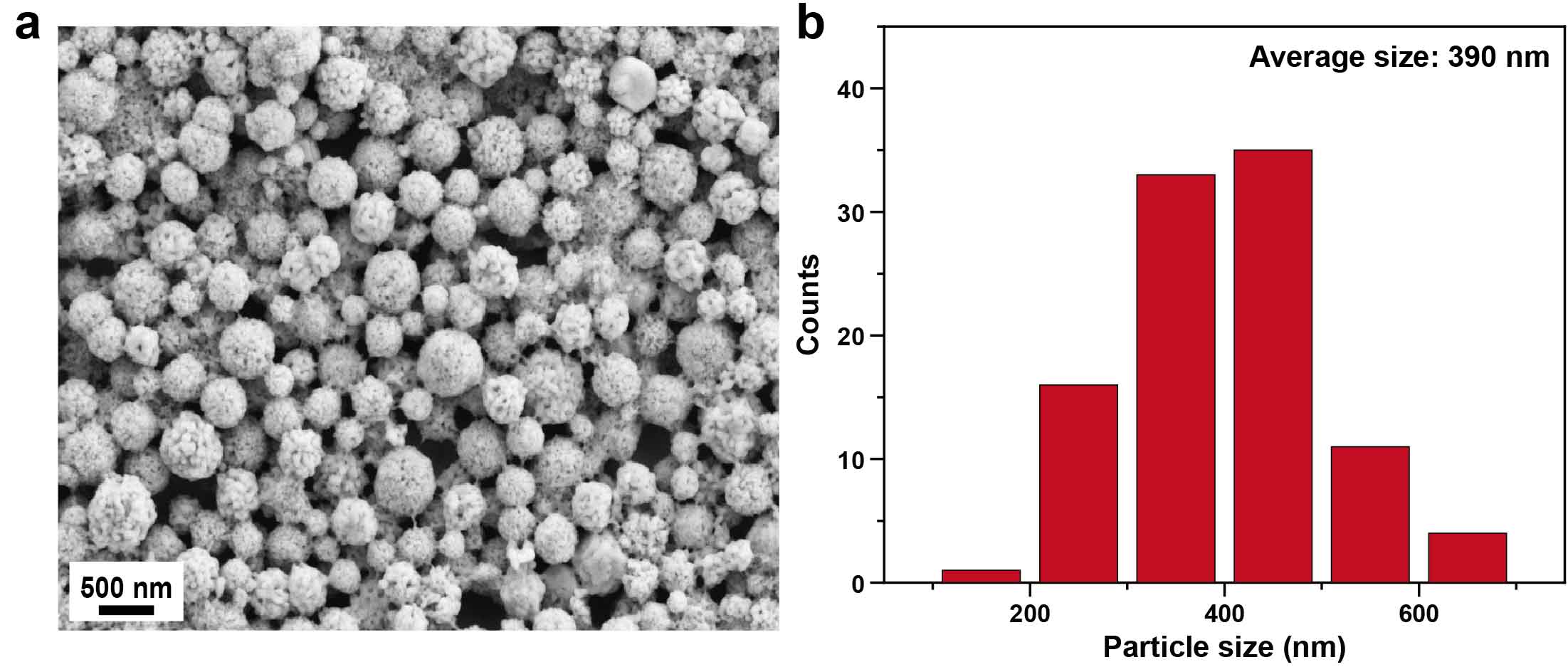
**

**Figure S7. Characterization of of Cu_4_I_6_(pr-ted)_2_ microparticles prepared by a room-temperature injection method.** **(a)** SEM image and **(b)** size distribution graph.

**
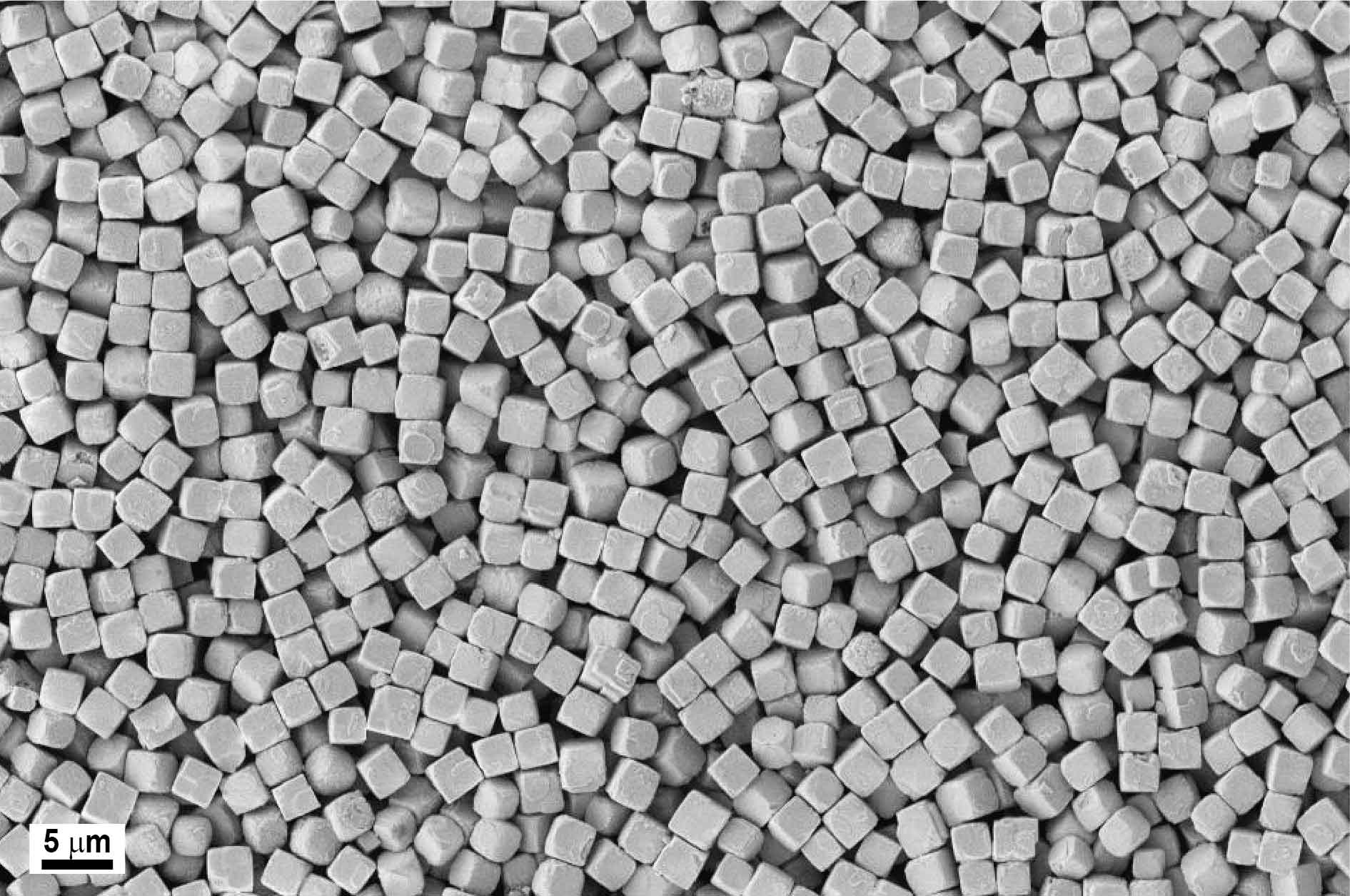
**

**Figure S8. Study of influence of calcinations on the morphology of Cu_4_I_6_(pr-ted)_2_ microcubes.** SEM image of Cu_4_I_6_(pr-ted)_2_ microcubes after calcination at 200 °C for 1.5 h in a nitrogen atmosphere.

**
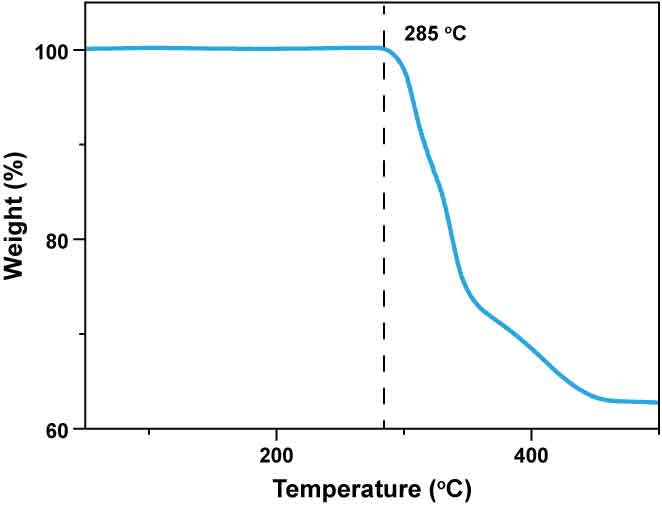
**

**Figure S9. The thermogravimetric analysis of Cu_4_I_6_(pr-ted)_2_ microcubes.** The results showed that Cu_4_I_6_(pr-ted)_2_ microcubes display high stability because of no degradation was observed when temperature lower than 285 ^o^C.

**
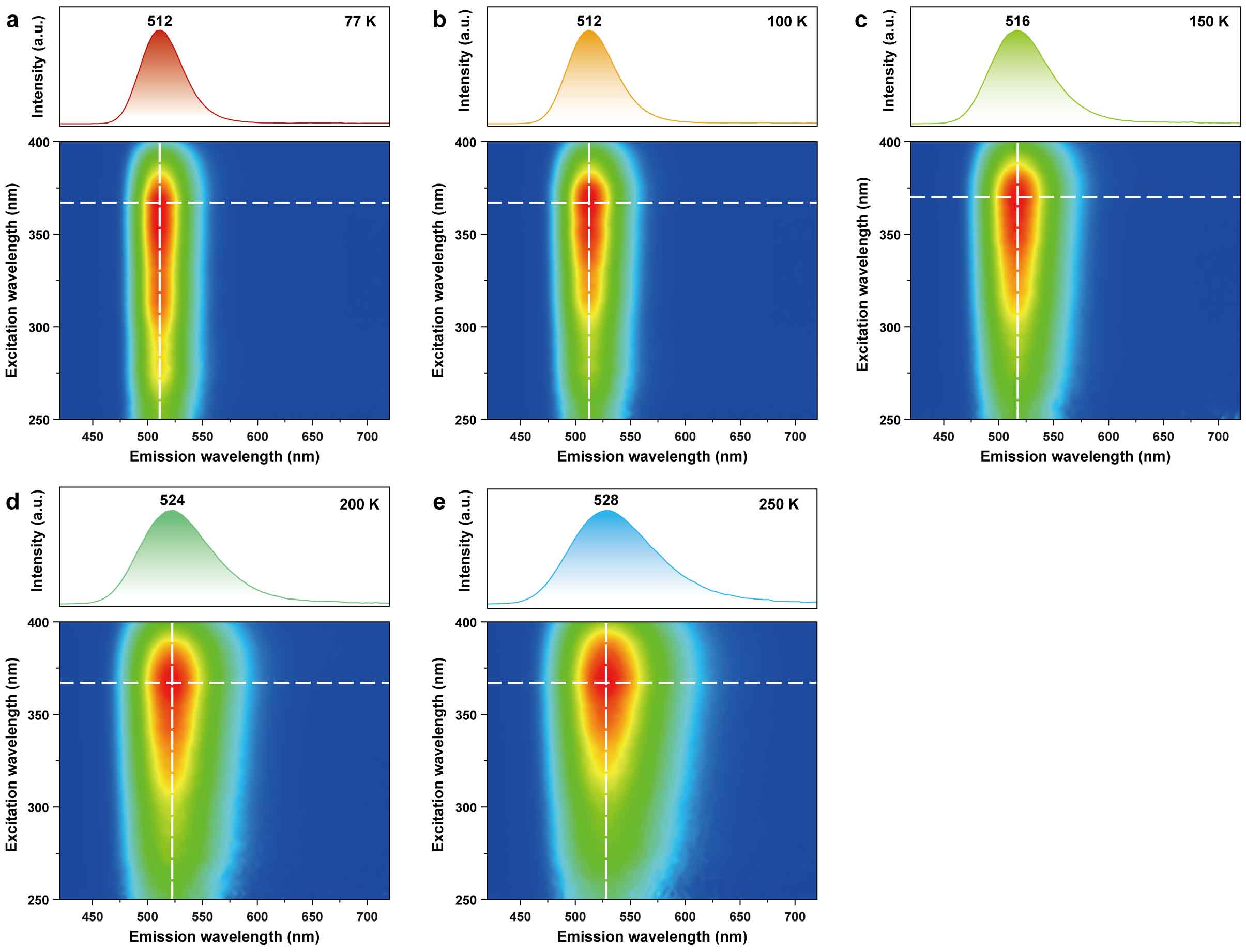
**

**Figure S10. Excitation-photoluminescence mapping of Cu_4_I_6_(pr-ted)_2_ microcubes at different temperatures.** A narrowing of the emission profile was observed as the temperature decreased, accompanied by a slight blue shift in the emission band. These observations were attributed to reduced structural torsion and an increased bandgap between the CBM and VBM of the Cu_4_I_6_(pr-ted)_2_ microcubes at low temperatures.

**
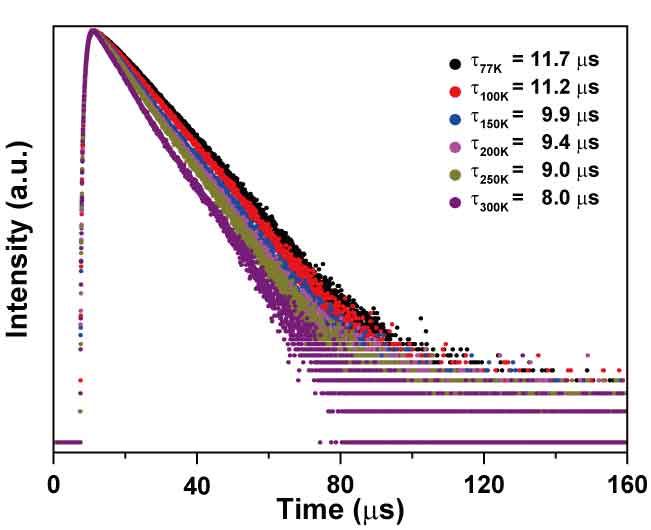
**

**Figure S11. Temperature-dependent decay curves of the green emission of Cu_4_I_6_(pr-ted)_2_ microcubes.** The lifetime was found to increase at low temperatures, suggesting that the green emission originates from the 3CC excited states with phosphorescence.

**
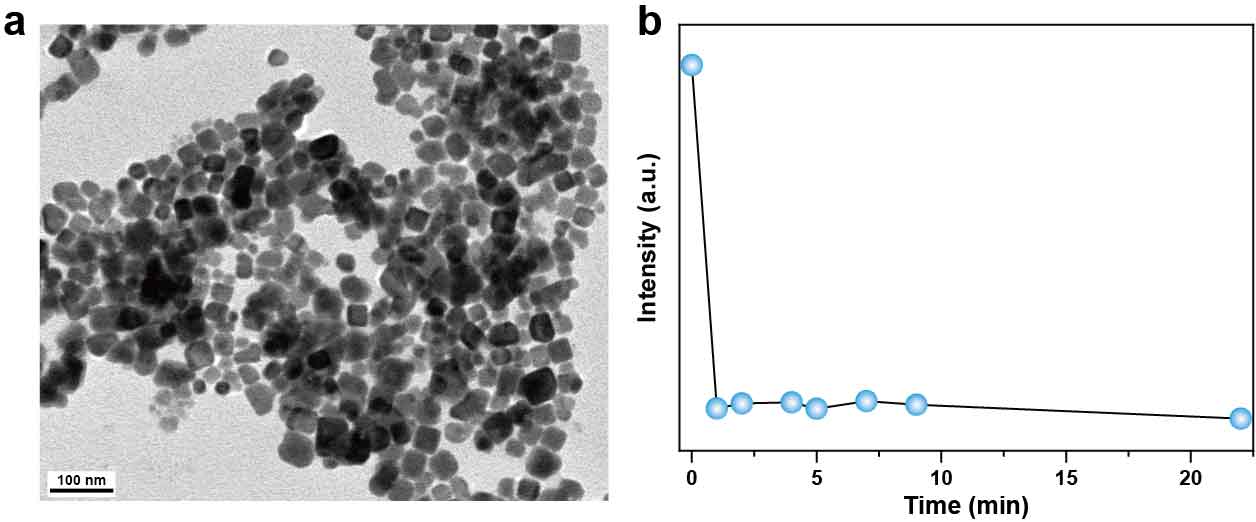
**

**Figure S12. Water resistance test of CsPbBr_3_ nanoparticles.** **(a)** TEM image of CsPbBr_3_ nanoparticles and **(b)** the change in emission intensity (544 nm) of a colloidal solution of CsPbBr_3_ nanoparticles after the addition of a small amount of water (3.2% volume).

**
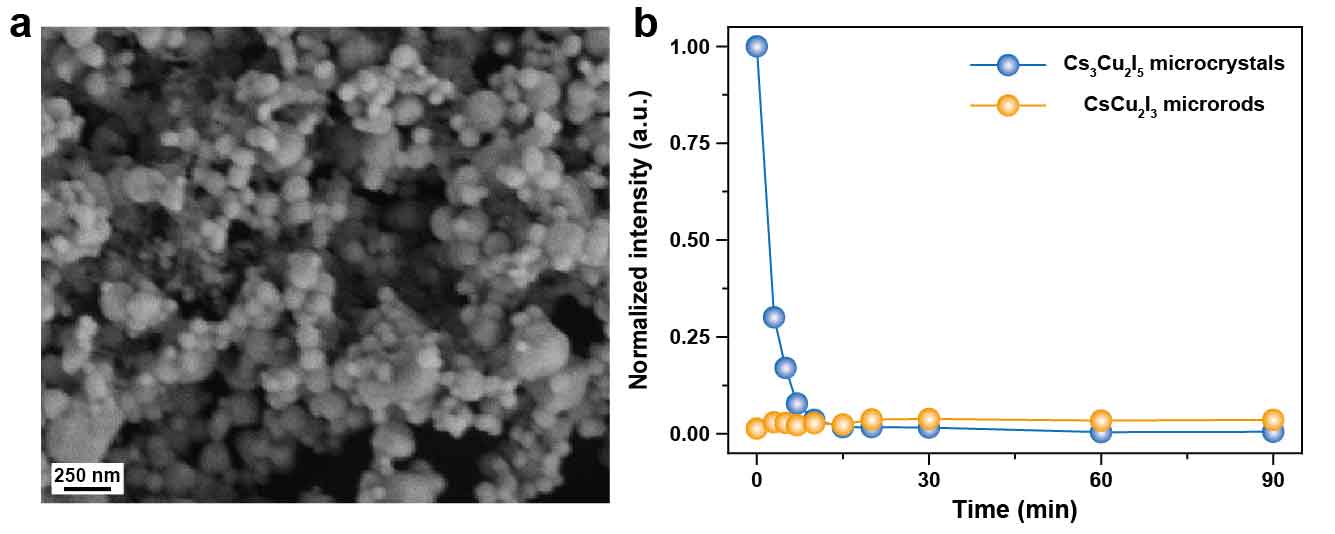
Figure S13. Water resistance test of Cs_3_Cu_2_I_5_ microparticles.** **(a)** SEM image of Cs_3_Cu_2_I_5_ microparticles and **(b)** the evolution of the emission intensity (459 nm) of a colloidal solution of Cs_3_Cu_2_I_5_ microparticles after the addition of a small amount of water (3.2 v%). Note that the orange curve shows the evolution of a new emission at 580 nm due to the transformation of Cs_3_Cu_2_I_5_ to CsCu_2_I_3_ in the presence of water.

**
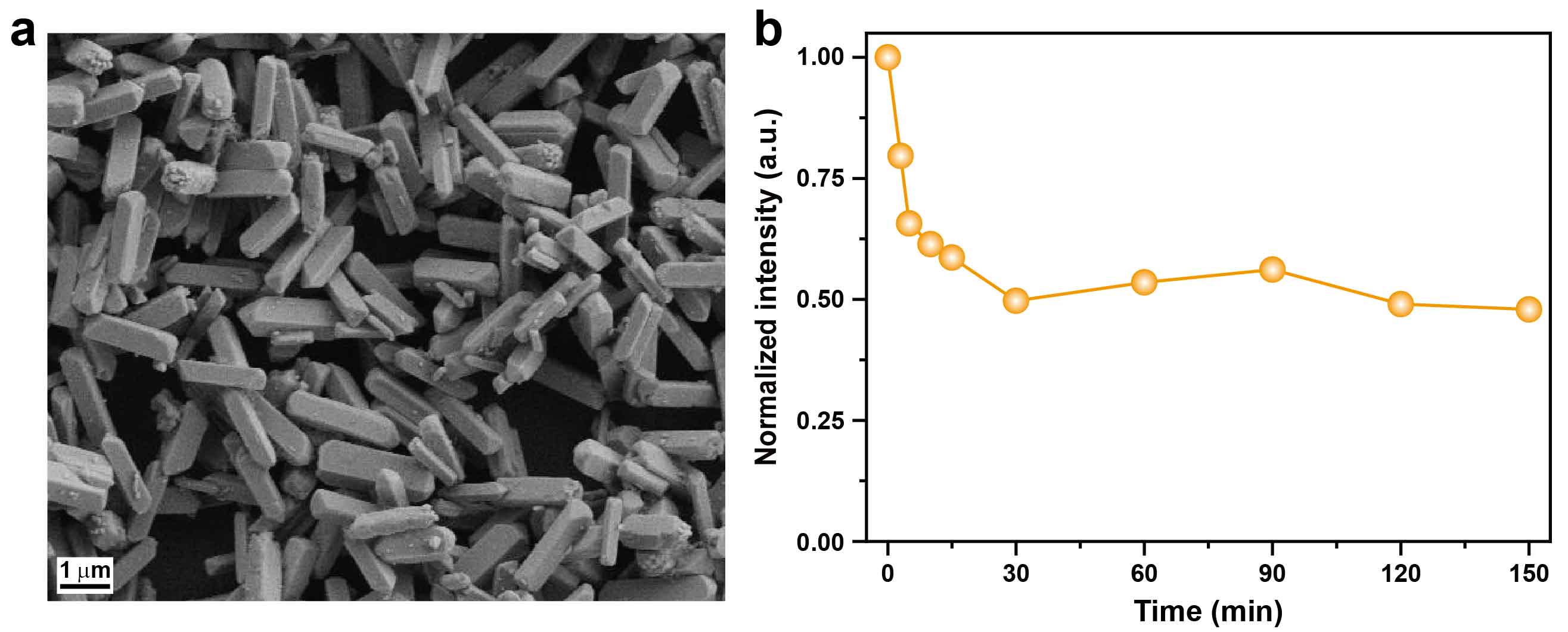
**

**Figure S14. Water resistance test of CsCu_2_I_3_ microparticles.** **(a)** SEM image of CsCu_2_I_3_ microrods and **(b)** evolution of the emission intensity at 580 nm of a colloidal solution of CsCu_2_I_3_ microrods after the addition of a small amount of water (3.2 v%).

**
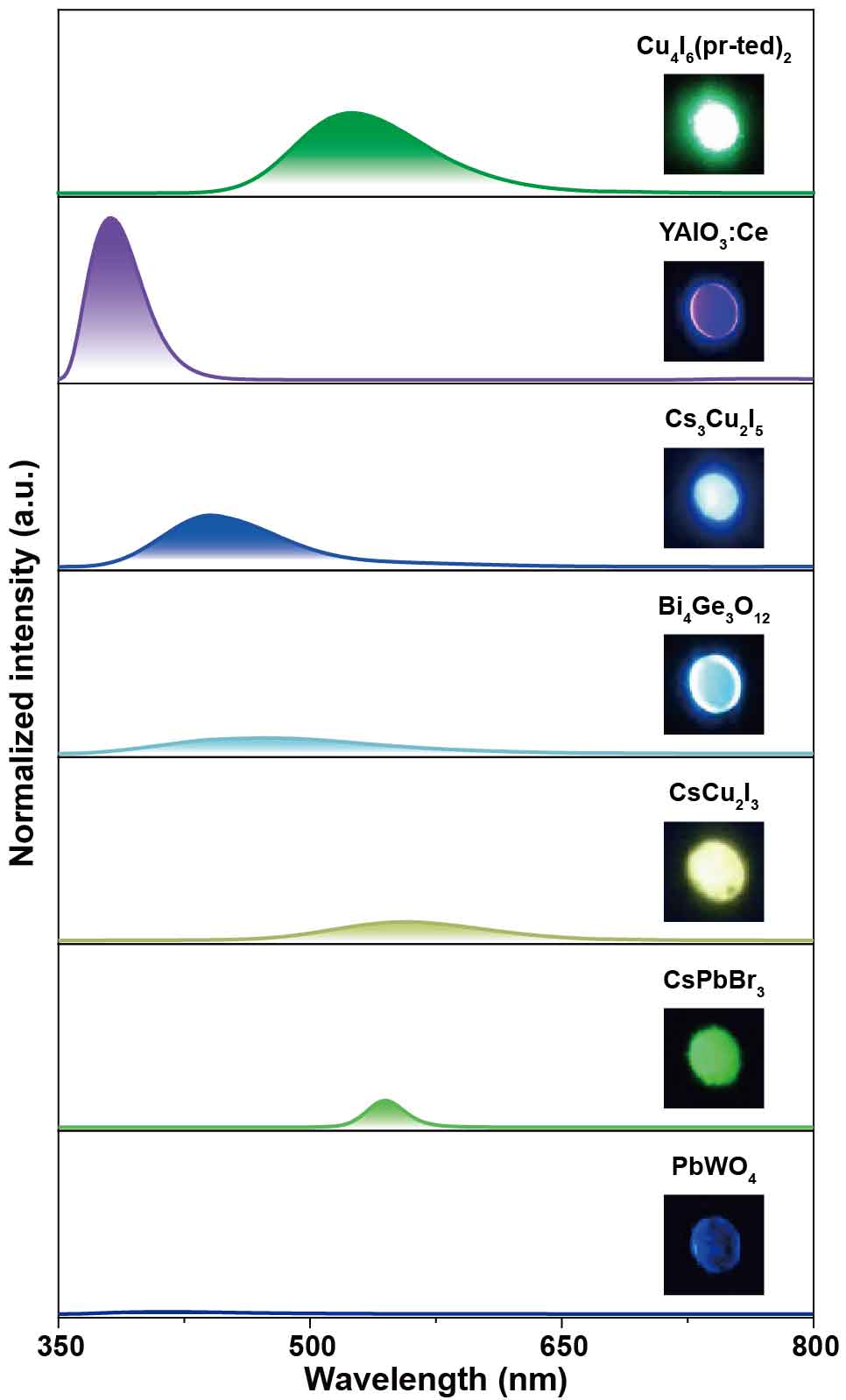
**

**Figure S15. Radioluminescence comparison study.** Radioluminescence profiles of YAlO_3_:Ce, Bi_4_Ge_3_O_12_, PbWO_4_ single crystals, and films of Cu_4_I_6_(pr-ted)_2_ microcubes, Cs_3_Cu_2_I_5_ microparticles, CsCu_2_I_3_ microrods, and CsPbBr_3_ nanoparticles. Note that the emission was normalized with the peak height of Cu_4_I_6_(pr-ted)_2_ microcubes, and the operation voltage of the X-ray tube was fixed at 60 kV.

**
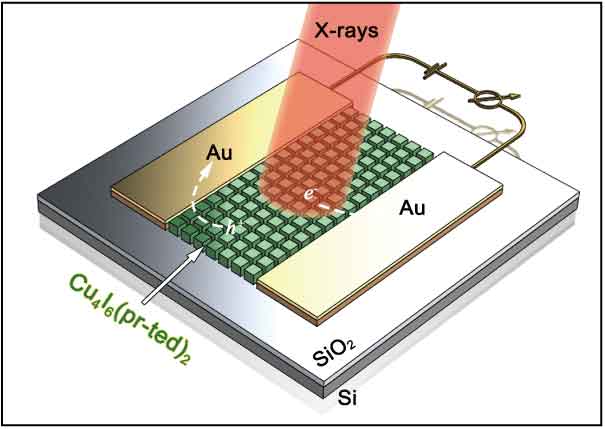
**

**Figure S16. Schematic of the device structure for probing X-ray-stimulated photocurrent.** The measured curve showed a higher current output upon X-ray irradiation at a dose rate of 3.4 mGy_air_ s^-1^, suggesting the conversion of X-rays to visible photons in Cu_4_I_6_(pr-ted)_2_ microcubes occurs through the formation of X-ray-induced charge carriers.

**
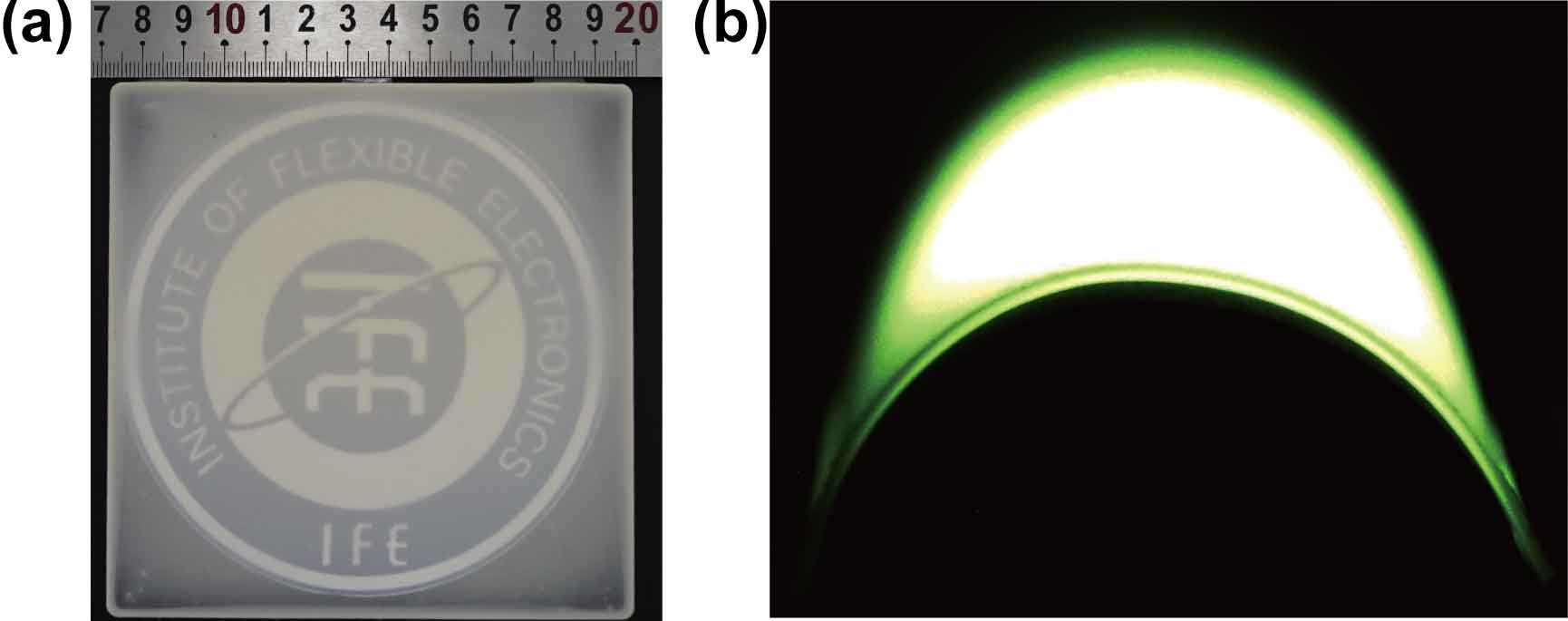
**

**Figure S17. Transparency and flexibility test of PDMS film doped with Cu_4_I_6_(pr-ted)_2_ microcubes. (a)** Photographs of a Cu_4_I_6_(pr-ted)_2_ microcube (5 wt%)-doped PDMS film (13 x 13 cm^2^) under natural light and **(b)** under X-ray irradiation (b).


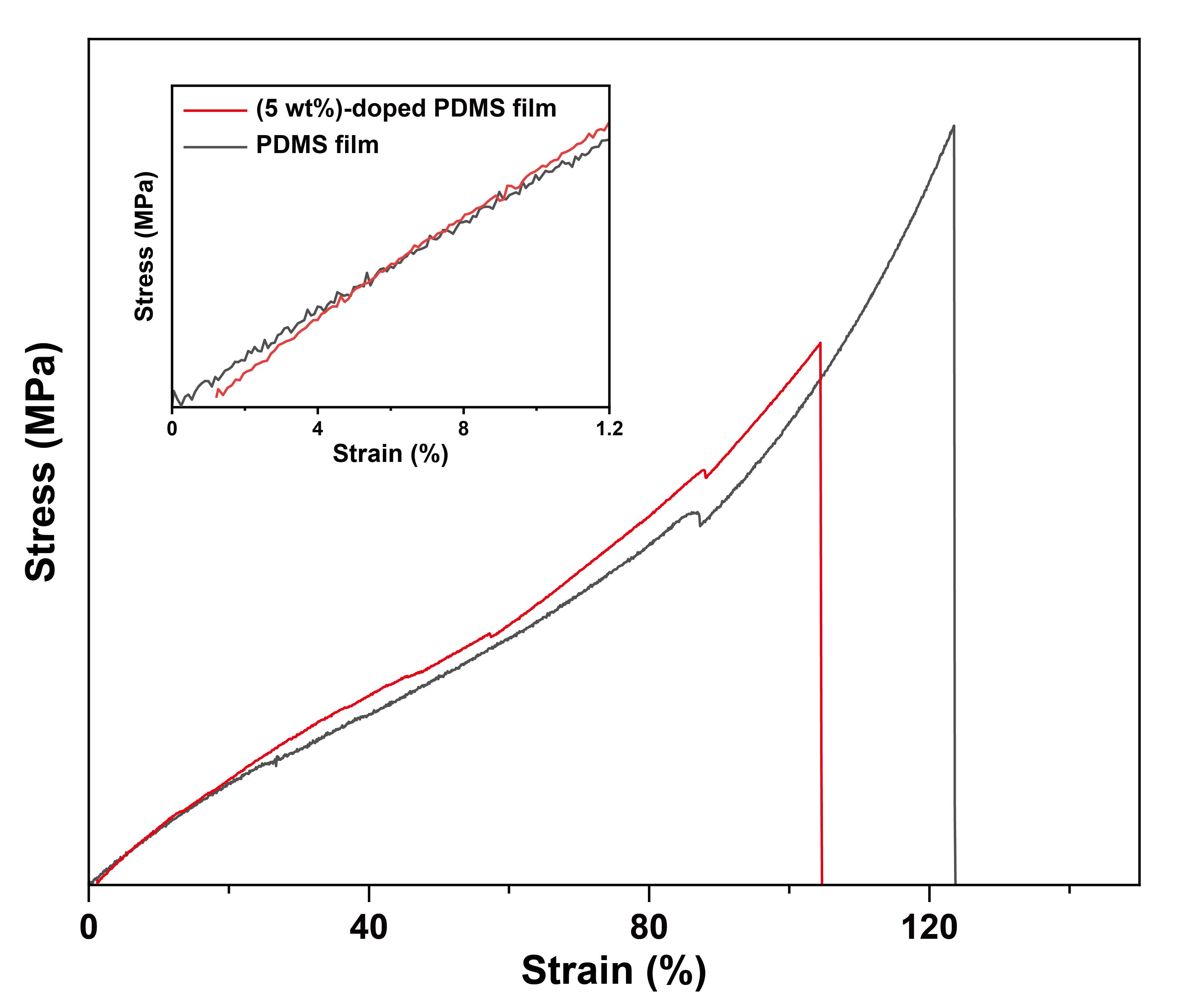


**Figure S18. Mechanical test of PDMS film doped with Cu_4_I_6_(pr-ted)_2_ microcubes.** Comparison of stress-strain curves between pure PDMS film and PDMS film doped with 5 wt% Cu_4_I_6_(pr-ted)_2_ microcubes.

**
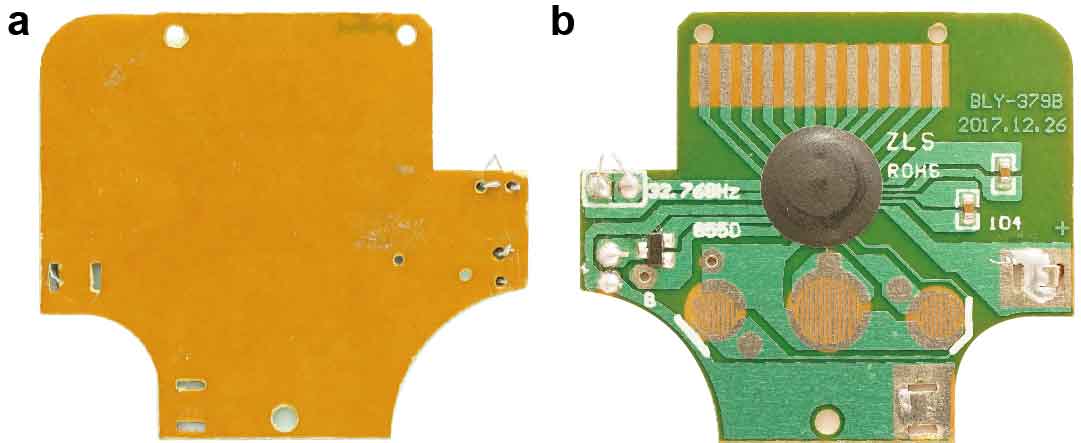
**

**Figure S19. The model used for X-ray imaging study.** Photographs of the back **(a)** and front **(b)** sides of a timer-printed circuit board.

**
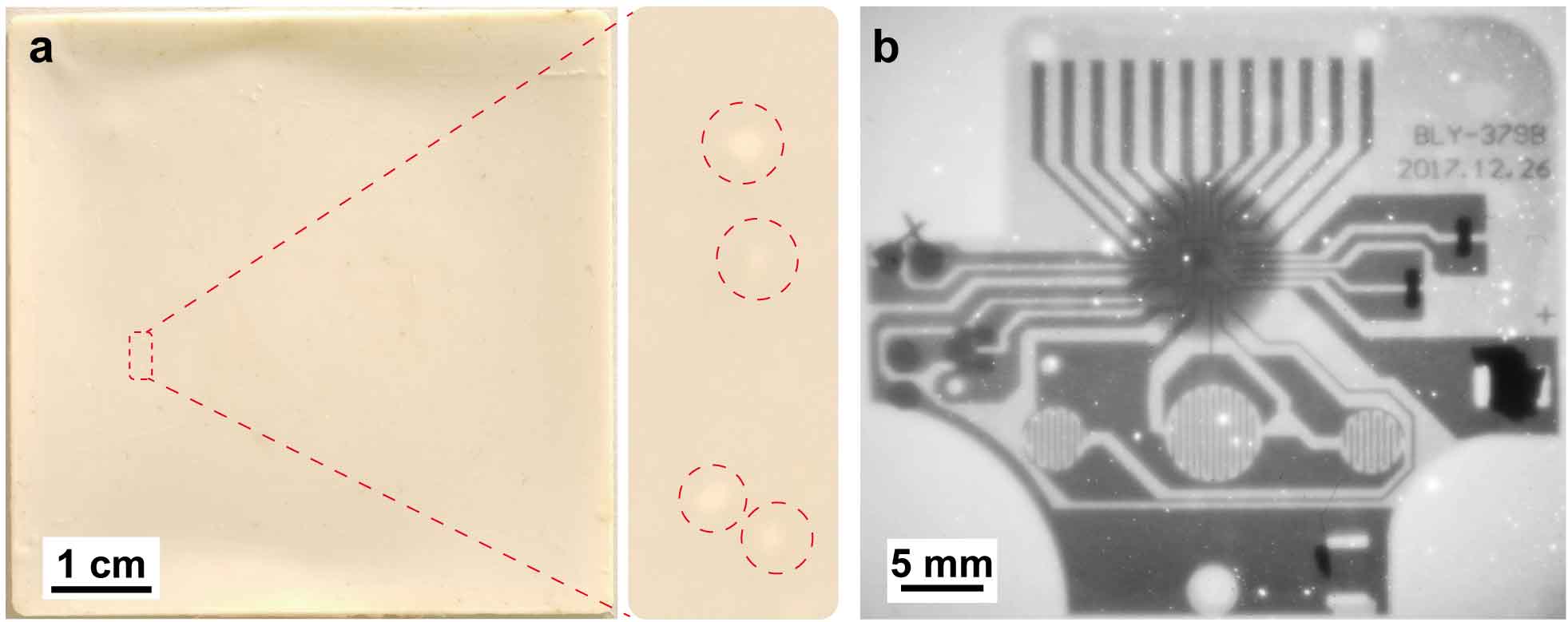
**

**Figure S20. Characterization and X-ray imaging of PDMS film doped with irregular Cu_4_I_6_(pr-ted)_2_ microparticles. (a)** Photograph of a PDMS film doped with irregular Cu_4_I_6_(pr-ted)_2_ microparticles at 5 wt%. **(b)** X-ray image of a timer-printed circuit board using the PDMS film.


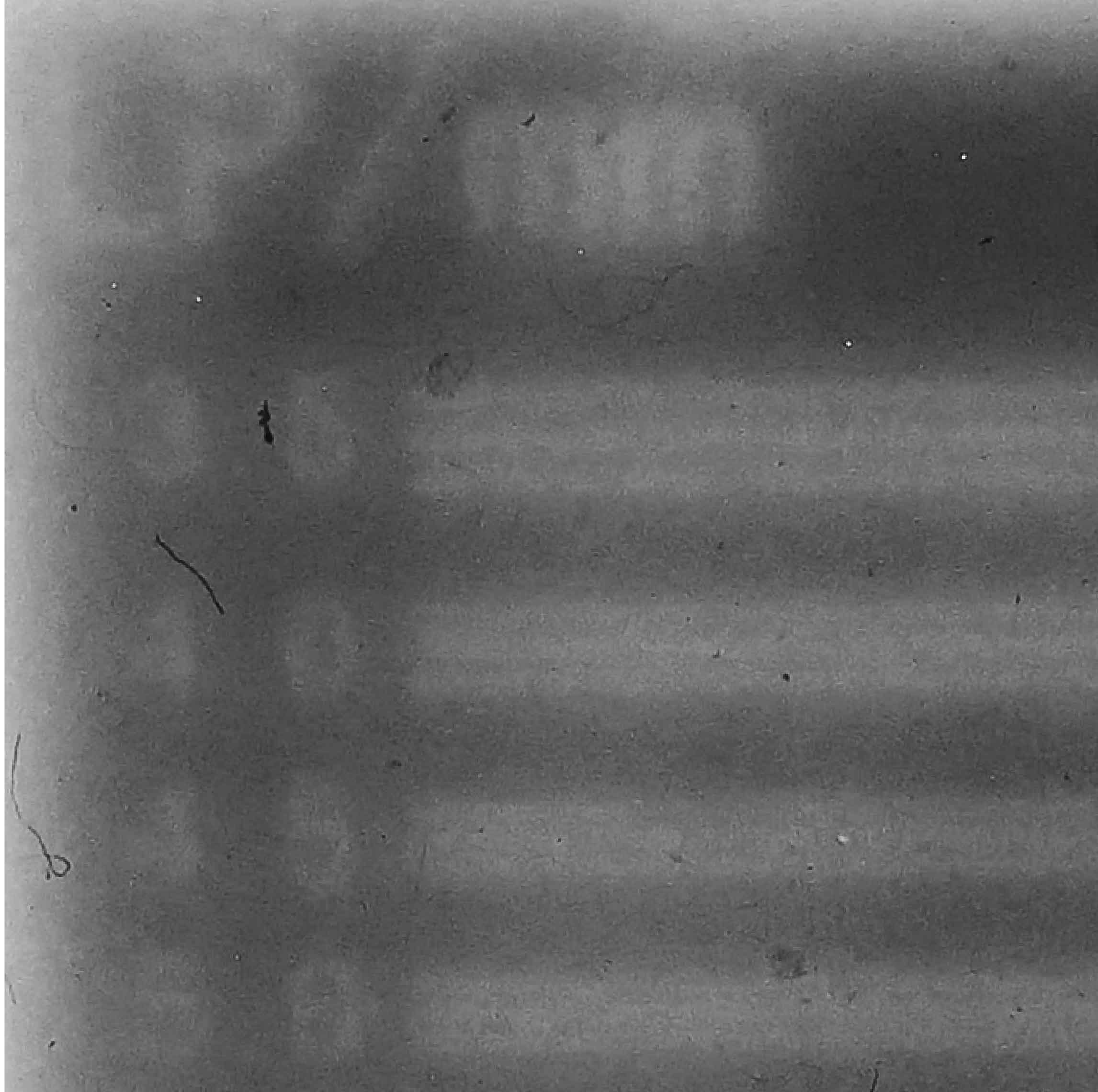


**Figure S21. Thickness-dependent X-ray imaging of PDMS film doped with Cu_4_I_6_(pr-ted)_2_ microcubes.** X-ray imaging of a standard X-ray pattern plate using a PDMS film (thickness: 1 mm) doped with 5 wt% Cu_4_I_6_(pr-ted)_2_ microcubes as a scintillation screen.


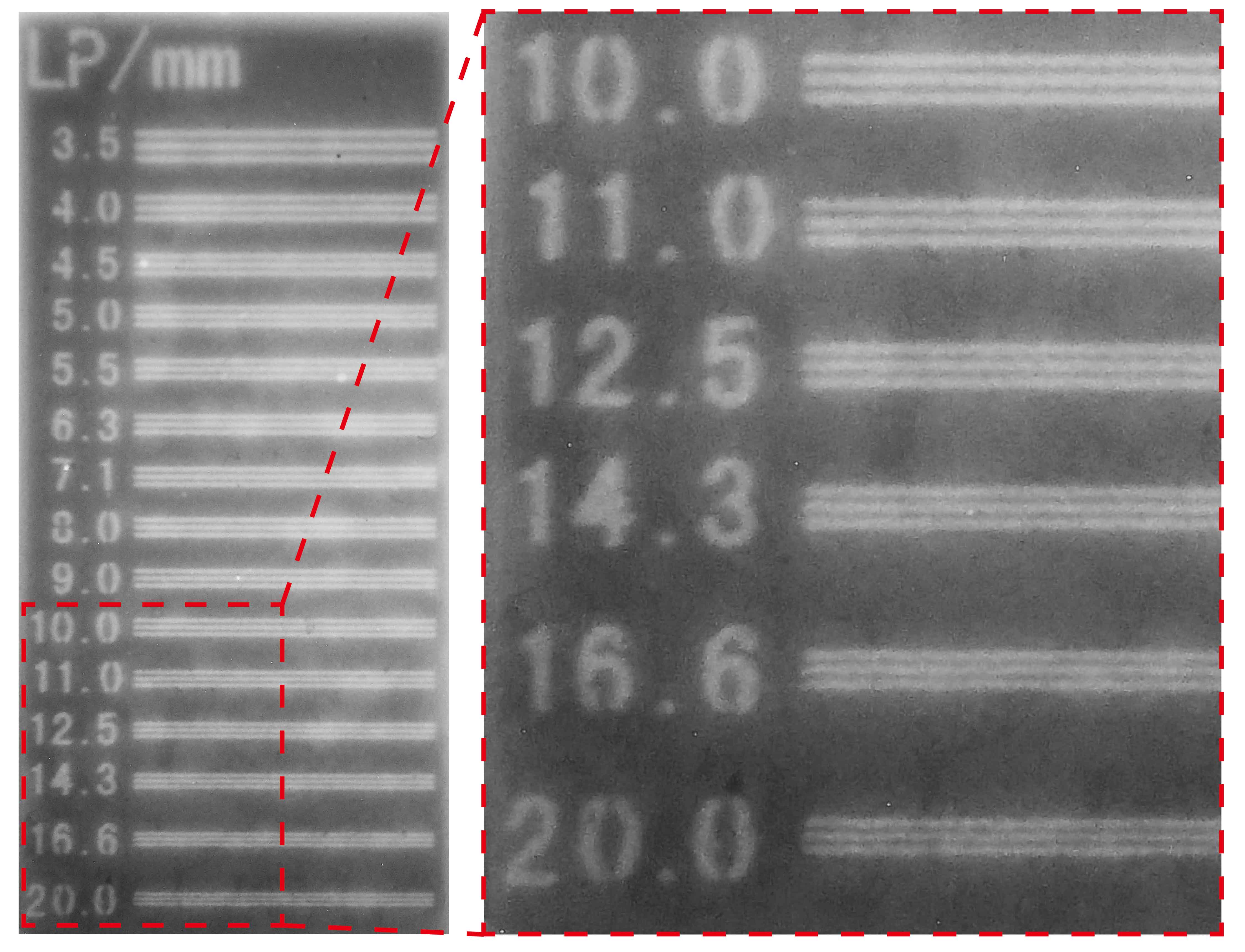


**Figure S22. Robustness test of PDMS film doped with Cu_4_I_6_(pr-ted)_2_ microparticles for X-ray imaging.** X-ray imaging of a standard pattern plate using a PDMS film (thickness: 0.5 mm) doped with 5.0 wt% Cu_4_I_6_(pr-ted)_2_ microcubes as scintillation screen after 2000 cycles of cyclic bending.

**
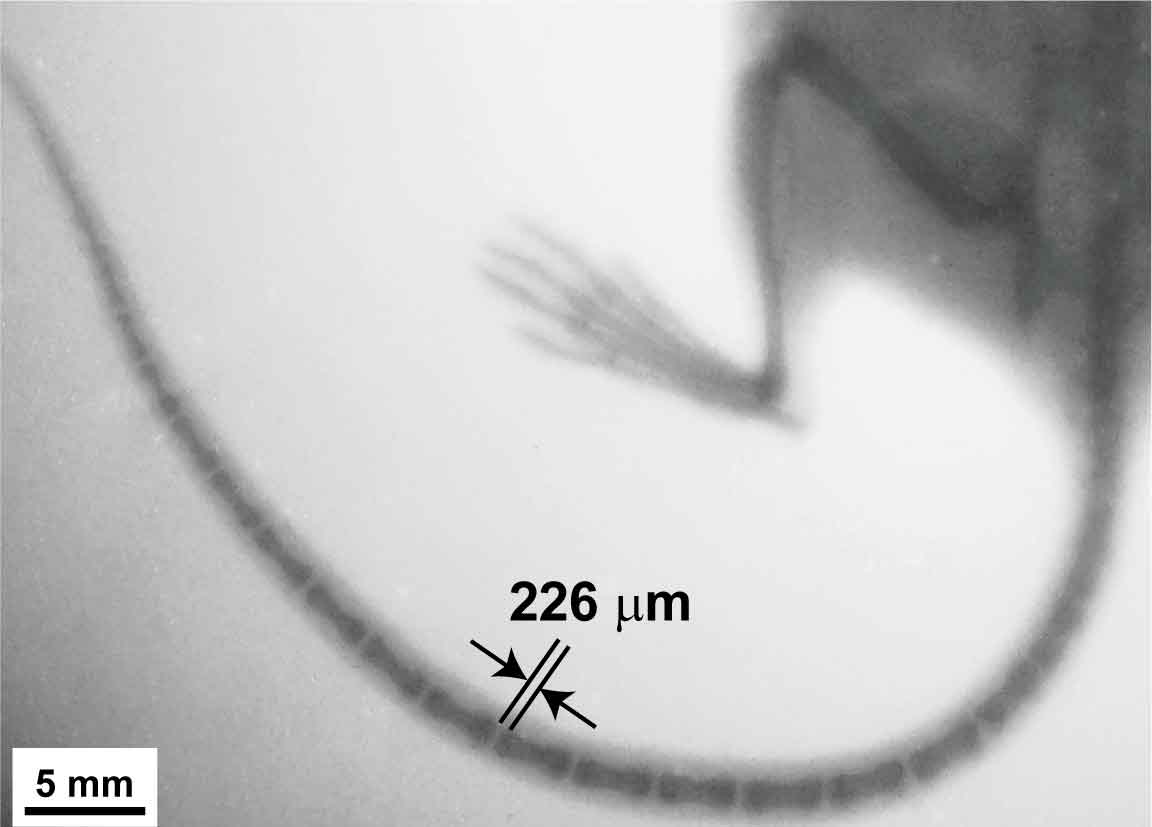
**

**Figure S23. High-resolution X-ray imaging of** **a nude mouse.** Enlarged X-ray image of the left hind limb and tail region of a mouse shows a clear interbone spacings of 226 μm.


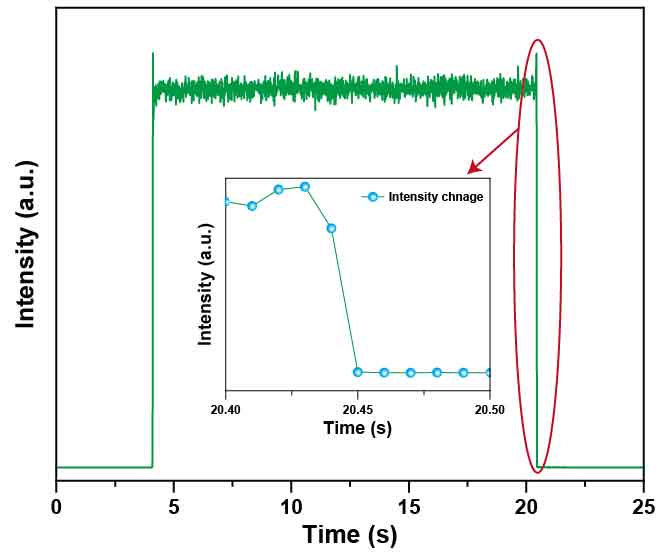


**Figure S24. The decay of the radioluminescence of Cu_4_I_6_(pr-ted)_2_ microcube after ceasing X-ray excitation.** Note that the radioluminescence decreased to a background level within 10 ms, suggesting the potential application of the Cu_4_I_6_(pr-ted)_2_ microcubes for dymanic X-ray imaging.

**
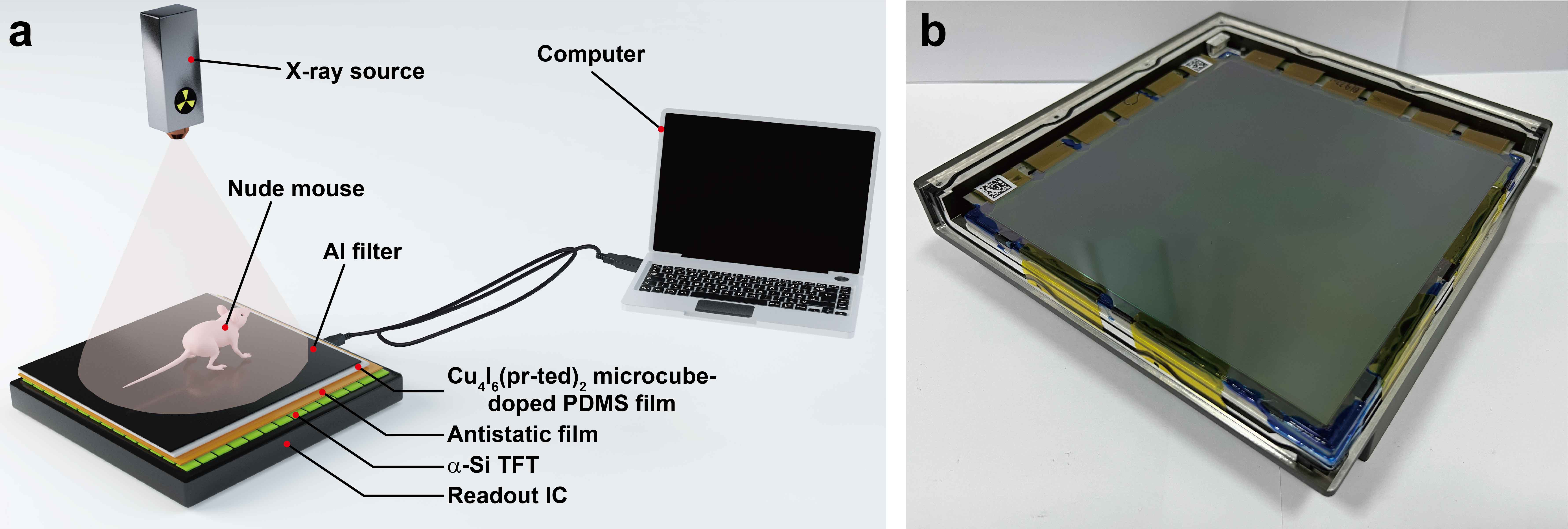
**

**Figure S25. Dynamic X-ray imaging study. (a)** Schematic of dynamic X-ray imaging of a nude mouse using a customized flat-panel detector and **(b)** a photograph of the detector without a scintillating film.

**IX. Supplementary Tables**

**Table S1.** Physical parameter comparison of Cu_4_I_6_(pr-ted)_2_ microcubes and other commercially available and reported scintillating materials

| Materials | Effective atomic number | Bandgap (eV) | PLQY (%) | Reference |
| --- | --- | --- | --- | --- |
| Cu_4_I_6_(pr-ted)_2_ microcubes | 46.5 | 2.72 | 97.1 | - |
| YAlO_3_:Ce crystal | 33.5 | - | - | 14 |
| Cs_3_Cu_2_I_5_ microcrystals | 52.4 | 2.60 | 49.2 | 2 |
| Bi_4_Ge_3_O_12_ crystal | 75.2 | 4.20 | - | 15 |
| CsCu_2_I_3_ microrods | 50.9 | 2.15 | 5 | 3 |
| CsPbBr_3_ QDs | 65.9 | 2.22 | 58.6 | 1 |
| PbWO_4_ crystal | 75.6 | 3.20 | - | 16 |

**Table S2.** Radioluminescence comparison of Cu_4_I_6_(pr-ted)_2_ microcubes and other commercially available and reported scintillating materials

| Materials | FWHM (nm) | Normalized emission area (a.u.) |
| --- | --- | --- |
| Cu_4_I_6_(pr-ted)_2_ microcubes | 89.907 | 100% |
| YAlO_3_:Ce crystal | 37.27 | 82.5% |
| Cs_3_Cu_2_I_5_ microcrystals | 80.025 | 63.1% |
| Bi_4_Ge_3_O_12_ crystal | 139.512 | 29.3% |
| CsCu_2_I_3_ microrods | 104.237 | 28.1% |
| CsPbBr_3_ QDs | 26.489 | 10.1% |
| PbWO_4_ crystal | 90.664 | 2.75% |

**X. References**

1. Chen, Q. et al. All-inorganic perovskite nanocrystal scintillators. *Nature* **561**, 88–93 (2018).
2. Lian, L. et al. Photophysics in Cs_3_Cu_2_X_5_ (X = Cl, Br, or I): Highly Luminescent Self-Trapped Excitons from Local Structure Symmetrization. *Chem. Mater.* **32**, 3462–3468 (2020).
3. Chen, P. et al. Colloidal Synthesis and Optical Properties of All-Inorganic Low-Dimensional Cesium Copper Halide Nanocrystals. *Angew. Chem.* **131**, 16233–16237 (2019).
4. Xie, M. et al. Highly efficient sky blue electroluminescence from ligand-activated copper iodide clusters: Overcoming the limitations of cluster light-emitting diodes. *Sci. Adv.* **5**, eaav9857 (2019).
5. Kobayashi, A., Kato, M. Stimuli-responsive Luminescent Copper(I) Complexes for Intelligent Emissive Devices. *Chem. Lett.* **46**, 154–162 (2017).
6. Chen, X. et al. X-ray-activated nanosystems for theranostic applications. *Chem. Soc. Rev.* **48**, 3073–3101 (2019).
7. Yanagida, T. Inorganic scintillating materials and scintillation detectors. *Proc. Jpn. Acad., Ser. B* **94,** 75–97 (2018).
8. Liu, X. et al. Probing the nature of upconversion nanocrystals: instrumentation matters. *Chem. Soc. Rev.* **44**, 1479–1508 (2015).
9. Perdew, J. P., Bieron, K., Ernzerhof, M. Generalized gradient approximation made simple. *Ernzerhof M. Phys. Rev. Lett.* **77**, 3865 (1996).
10. Heyd, J., Scuseria, G. E., Ernzerhof M. Hybrid functionals based on a screened Coulomb potential. *J. Chem. Phys.* **118**, 8207 (2003).
11. Heyd, J., Scuseria, G. E., Ernzerhof M. Erratum: “Hybrid functionals based on a screened Coulomb potential”. Chem. Phys.118, 8207 (2003)]. *J. Chem. Phys.* **124**, 219906 (2006).
12. Kresse, G. Hafner, J. Ab initio molecular dynamics for open-shell transition metals. *Phys. Rev. B* **48**, 13115 (1993).
13. Kresse, G., Furthmuller, J. Efficiency of ab-initio total energy calculations for metals and semiconductors using a plane-wave basis set. *Comput. Mater. Sci*. **6**, 15-50 (1996).
14. Laguta, V. et al. Incorporation of the Ce^3+^ activator ions in LaAlO_3_ crystals: EPR and NMR study. *J. Solid State Chem.* **313**, 123295 (2022).
15. Jellison, G. E. et al. Optical properties of bismuth germanate. *J. Appl. Phys*. **107**, 013514 (2010).
16. Fabeni, P. et al. Luminescence and photo-thermally stimulated defects creation processes in PbWO_4_ crystals doped with trivalent rare-earth ions. *J. Lumin.* **136**, 42–50 (2013).
